# Morphology, development stages, and phylogeny of the *Rhabditolaimus ulmi* (Nematoda: Diplogastridae), a phoront of the bark beetle *Scolytus multistriatus* from the elm *Ulmus glabra* Huds. in Northwest Russia

**DOI:** 10.21307/jofnem-2021-025

**Published:** 2021-03-11

**Authors:** Alexander Y. Ryss, Kristina S. Polyanina, Sergio Álvarez-Ortega, Sergei A. Subbotin

**Affiliations:** 1 Zoological Institute of the Russian Academy of Sciences, Universitetskaya Naberezhnaya 1, St Petersburg, 199034, Russia; 2 Departamento de Biología y Geología, Física y Química Inorgánica, Universidad Rey Juan Carlos, Campus de Móstoles, 28933, Madrid, Spain; 3 Plant Pest Diagnostic Center, California Department of Food and Agriculture, 3294 Meadowview Road, Sacramento, CA, 95832; 4 Center of Parasitology of A. N. Severtsov Institute of Ecology and Evolution of the Russian Academy of Sciences, Moscow, 117071, Russia

**Keywords:** Bark beetles, Dauer, Dutch elm disease, Life cycle, Molecular phylogeny, Morphology, Ovoviviparity, Phoresis, Symbiosis, Tabular key, Transmissive infections

## Abstract

The nematode *Rhabditolaimus ulmi* was found in galleries, adults, and larvae of *Scolytus multistriatus*, the vector of the Dutch elm disease, in St. Petersburg parks. This nematode co-occurred with *Bursaphelenchus ulmophilus*, which is another phoretic partner of *S. multistriatus*. Nematodes were cultured on the fungus *Botryotinia fuckeliana* in potato sugar agar (PA) and used for morphological analyses of adults, juveniles, eggs, and dauers. Nematode females showed a didelphic female genital tract rather than a monoprodelphic gonad as reported in the original description. Male bursa peloderan, caudal papillae include three preanal pairs and one precloacal unpaired papillae; seven postanal papilla pairs, among which one is pore-like and possibly the phasmid homolog, one subdorsal, and a pair of three closely situated posteriorly at bursa alae. The juvenile stages differ in size and structure of their sexual primordia. Sex of juveniles may be identified from the third stage. The dauer juvenile is a phoretic third juvenile stage (DJ3), which enters and remains localized in the buccal cavity of beetle adults and last-instar larvae and also under the elytra and in the ovipositor’s cavity of pupae and imagoes. The first molt J1-J2 occurred inside the eggshell. Adult females laid eggs in early stages of embryonic development or containing molted J2. The propagative non-phoretic J2 inside the egg and J3 have a long and well-developed median bulb. The phoretic dauer DJ3 has a small spherical bulb like the J1 juvenile within the egg. In a sterile fungal culture, the nematodes feed on both mycelium and their unidentified ecto-symbiotic bacteria, located on nematode surface coat and multiplying in PA. Diagnosis and tabular key to the *Rhabditolaimus* species are given. Phylogenetic analysis of the D2-D3 of 28S rRNA gene sequences resulted in the Bayesian consensus tree with the highly supported clade of the *Rhabditolaimus* species.

Among wood- and bark-inhabiting nematodes, the fungal feeders in the family Aphelenchoididae, such as the pinewood nematode, *Bursaphelenchus xylophilus* (Nickle, 1970; Steiner and Buhrer, 1934) and the red ring nematode, *B. cocophilus* (Baujard, 1989; Cobb, 1919) have been studied extensively because of the devastating diseases they cause on pines and palms, respectively, and their spreading capabilities using insect vectors (Jones et al., 2013; Mota et al., 1999; Ryss et al., 2005). In the nematode fauna of declining trees, some species are vectored by bark and longhorn beetles (families Curculionidae and Cerambycidae). These nematode phoronts use their insect vectors to colonize live trees or for transmission to new decaying wood sites to feed on fungi or bacteria. The transmissive dispersal life stage of these nematodes is the dauer juvenile (DJ), which is morphologically different from the non-phoretic juveniles of the propagative generation inhabiting wood and bark. The ecological roles of many obligatory phoronts of the bark beetles belonging to the families Diplogastridae and Rhabditidae are not known.

Only two nematode species were found most consistently in a 2014 to 2020 survey of declining *Ulmus glabra* and *U. laevis* trees in St. Petersburg parks: *Bursaphelenchus ulmophilus* (Ryss et al., 2015) and *Rhabditolaimus ulmi* (Goodey, 1930; Susoy and Herrmann, 2012). The latter was originally described by Goodey (1930) and later redescribed by Rühm (1956) from Bavaria, Germany. However, the biology, morphology, and phylogeny of this species have not been well elucidated. Both species were detected in galleries of *Scolytus multistriatus* (Marsham, 1802), a bark beetle that is known to transmit Dutch elm disease (DED). Their dauer juveniles were also regularly collected in beetle adults, under elytra.

The objectives of this paper were to (i) describe and characterize morphologically all developmental stages of *R. ulmi*, including the dauer juvenile stage, (ii) describe *R. ulmi*’s ecological role in relation to its vector life cycle, and (iii) provide molecular characterization of *R. ulmi* and reconstruct phylogeny of the genus.

## Materials and methods

### Nematode samples

During 2014 to 2020 declining *Ulmus glabra* Huds. trees were surveyed for wood-inhabiting nematodes in parklands of eight St. Petersburg city districts: Admiralteisky, Vyborgsky, Vasileostrovsky, Krasnoselsky, Kirovsky, Moscowsky, Petrogradsky, and Primorsky. In total, 25 locations were selected for consistent sampling.

In previous years, all trees that showed symptoms of dieback were found infected by the fungus *Ophiostoma novo-ulmi* Brasier, the causal agent of DED and vectored by the *S. multistriatus* (Marsham, 1802) beetle (Kalko, 2008, 2009). The elm bark with beetle galleries, as well as beetle larvae, imago, and pupae were sampled for nematodes in all locations. In total, 75 bark samples with live adult beetles and larvae were taken. In February 2019, additional 15 wood and bark samples were also collected during the sanitary cutting of declining elms infected by DED in the park of the St. Petersburg Forestry and Technical University (GPS coordinates: 59.991923°N, 30.342697°E). Nematodes were extracted from these samples and then cultured on the fungus *Botryotinia fuckeliana* (de Bary) Whetzel, 1945 (= *Botrytis cinerea* Pers., 1794) on potato sugar agar media (PA).

### Nematode extraction and rearing

Nematodes were extracted from bark by Baermann’s funnel technique modified by Ryss (2015). To extract nematodes from beetles, the elytra of adults or a whole larvae or pupa were placed in a 0.5-ml drop of distilled water and incubated for 30 to 60 min to allow the nematodes to slowly move from insect body into the water. Cultures of *B. fuckeliana* fungus on the 2% potato sugar agar media (PA) were used for nematode rearing. In total, 50 g of fresh sliced potato tubers were boiled for 40 min in 200 ml of water, then smashed, and additionally boiled for 5 min, filtered through gauze. In total, 4 g of agar, 4 g of sugar, and 10 ml of glycerin were added to the hot solution, the volume was brought to 200 ml, and finally autoclaved at 3 atm and 130°C for 40 min. The hot mixture was poured into 6-cm Petri dishes, cooled down, and inoculated with mycelium of a strain of *B. fuckeliana*, which does not produce spores. After 5 to 7 days at 20°C, when a white mycelium lawn occupied the entire surface of the agar, 20 *R. ulmi* specimens that had been extracted from the bark and identified with a compound microscope, were transferred to the fungal culture. Separately, dauers taken from beetles were also reared in fungal cultures, reaching the maturity and then producing the nematodes of propagative generation.

After 10 to 14 days, nematodes had multiplied and consumed all the mycelium. Simultaneously, bacteria from the surface coat of the nematode body grew onto the agar medium. Nematode cultures were passaged 2 to 3 times until all the nematodes were *R. ulmi* at different stages of development: eggs, juveniles, and adults. Every 10 days, cultures were transferred to new PA media with fungus. After every five cycles in fungal culture, nematodes were passed through a superficially sterilized (30 min in a 1.0% NaOCl household bleach solution) elm branch for one month, followed again by culture on *B. fuckeliana*. Cultures of the nematode isolates in Petri dishes can be stored in a refrigerator at + 8°C for up to six months without recultivation. Nematodes obtained from these cultures were used for morphological analysis.

### Morphological study

#### Fixation and mounting

Nematodes were fixed with hot TAF (2 ml of triethanolamine, 10 ml of formalin, and 90 ml of distilled water) in a water bath (Ryss, 2017a), processed in a glycerol-water series, and mounted in the permanent collection slides using the method described by Ryss (2003, 2017b). Some juveniles fixed in TAF and processed to anhydrous glycerin, were stained with methylene blue stain by adding a 10-µl drop of saturated water solution of methylene blue to a drop of glycerin with the suspended nematode juveniles, on a 75 × 25-mm slide. After 12 hr, the stained suspension was covered with a 20 × 20-mm coverslip previously sealed on its edges with a thin film of glycerin jelly. After quick 60°C heating on a hot plate, the drop spread reaching edges of the coverslip; thus, the suspension was flattened and fixed with a glycerin jelly frame. The stained juveniles were studied and photographed using a Leica compound microscope. Following their examination and identification, a few specimens preserved in glycerin were selected for scanning electron microscopy (SEM) observations. The nematodes were hydrated in distilled water, dehydrated in a graded ethanol and acetone series, critical point-dried, mounted on stubs, coated with gold, and observed with a Zeiss Merlin microscope (Álvarez-Ortega and Peña-Santiago, 2016). The Adobe Photoshop CS2 software was used to adjust the contrast of photos, the ImageJ software for measurements (Collins, 2007), and MS Excel for calculations and statistical processing. Terminology of Fürst von Lieven and Sudhaus (2000) was used to describe the pharynx and stoma structures.

#### Nematode localization in beetles

The beetle larvae, pupae, and imagoes were examined for presence and localization of nematode dauers using the stereomicroscope LOMO MBS-10. Surface deworming was used to check the adhesive strength between the nematode and vector. The insect larvae and adults were immersed for 45 min in a 5% solution of acetic acid and 0.02% chlorhexidine and then washed with water on a 200-µm sieve. Beetles and most nematode dauers remained alive and attached to insect mouth parts and cavities, while other nematode stages could not stay attached and were washed out. This test was aimed to identify places in the body of the vector where nematodes could remain viable under aggressive external influences.

#### DNA extraction, PCR, sequencing, and phylogenetic analysis

DNA was extracted from several pooled nematodes using proteinase K. PCR and sequencing protocols were as described by Tanha Maafi et al. (2003). The forward *Rhabditolaimus*_D2A-1 – (5′-GGC GAA GCG GAT AGA GTT GAC-3′) and reverse D3B (5′-TCG GAA GGA ACC AGC TAC TA-3′) primers were used for amplification of the D2-D3 expansion segments of 28S rRNA gene. Sequencing was done at Genewiz., Inc (CA, USA). A sequence was deposited in GenBank under the accession number: MW044951.

The D2-D3 expansion segments of 28S rRNA gene sequence of *R. ulmi* was aligned with sequences of other *Rhabditolaimus* and related genera (Kanzaki and Giblin-Davis, 2014; Kiontke et al., 2007; Susoy and Herrmann, 2012, Susoy et al., 2015; and others). The alignment was analyzed with Bayesian inference (BI) using MrBayes 3.1.2 (Ronquist and Huelsenbeck, 2003) using the GTR + I + G model. BI analysis was as described by Ryss et al. (2021).

## Results

### Detection of nematodes in trunks and branches

Of 75 samples, 20 were pieces of branches 20-cm long and 3 to 10 cm in diameter collected during sanitary cuttings. These twig segments are parts of the tree crowns contained the bark holes formed by beetles during maturation feeding. A red-brown ring in phloem was found in the cross section, which is typical for symptoms of DED. In bark, the fungal and plant-feeding nematodes *B. ulmophilus* and *Aphelenchoides* sp. were found, but not *R. ulmi*.

Other samples were 20 × 30-cm side parts of trunk segments, 3 to 6-cm thick, with the bark beetle galleries in the inner bark layer were consistently inhabited by both nematode species, *R. ulmi* and *B. ulmophilus*. In the period between February and June, the beetle larvae and imagoes with nematode dauers were present in the trunk samples. When the dauers from these samples were placed on the *B. fuckeliana* culture, they molted into preadult juveniles and then into adults of the transmissive generation, and then grew in cultures transforming to adults and juveniles of the propagative generations of two nematode species, *R. ulmi* and *B. ulmophilus*. The total number of trunk segments with living beetle larvae and adults was 25, whereas other 30 samples contained empty galleries from which the beetles had already emerged.

### Nematode culture

*Rhabditolaimus ulmi* successfully propagated in sterile plates seeded with *B. fuckeliana*. Observations of nematode inoculated fungal plates revealed that nematodes grab the mycelium by the head probolae and stoma. Unidentified bacteria were also observed on the surface body coat of nematodes in the agar media and in the intestine of the nematode, suggesting that the nematodes were feeding on both fungi and bacteria.

### Nematode localization in beetle

*Rhabditolaimus ulmi* nematodes were found under the elytra of beetle imagoes, in the ovipositor and buccal cavity, the latter containing the highest number of dauers. Dauers were observed in the buccal cavity and in developing ovipositor cavity of beetle pupae. In later stages beetle larvae nematodes were localized on the body surface and in the buccal cavity. When treated with deworming solutions, the nematodes were removed from the body surface but remained in the buccal cavity. In beetle adults and pupae nematodes remained also in the ovipositor cavity. Many of the beetles contained both *B. ulmophilus* and *R. ulmi*. The *B. ulmophilus* dauers were easily recognized by the stylet and very narrow body shape; they were completely flushed out when treated with deworming solutions.

## Description

### *Rhabditolaimus ulmi* (T. Goodey, 1930) Susoy and Herrmann, 2012

(Figs. 1-9).

**Figure 1:**
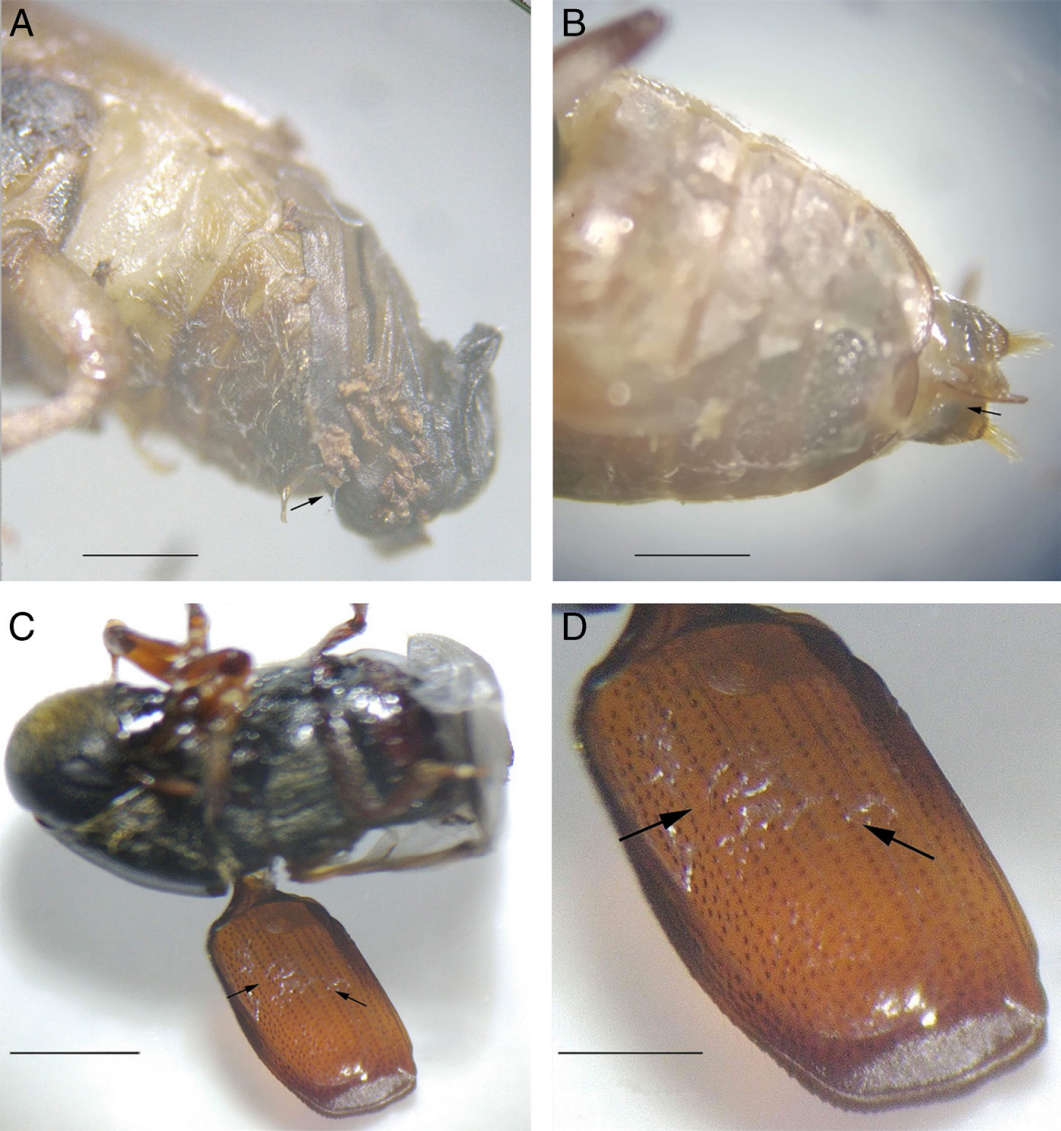
Localization of the *Rhabditolaimus ulmi* dauer juveniles in pupae (A, B) and imago (C, D) of the beetle vector *Scolytus multistriatus.* A – on the elytra primordia; B – at the oviposititor opening; C, D – on the elytrae of imago. Scale: 1 mm for D, 2 mm for rest.

**Figure 2:**
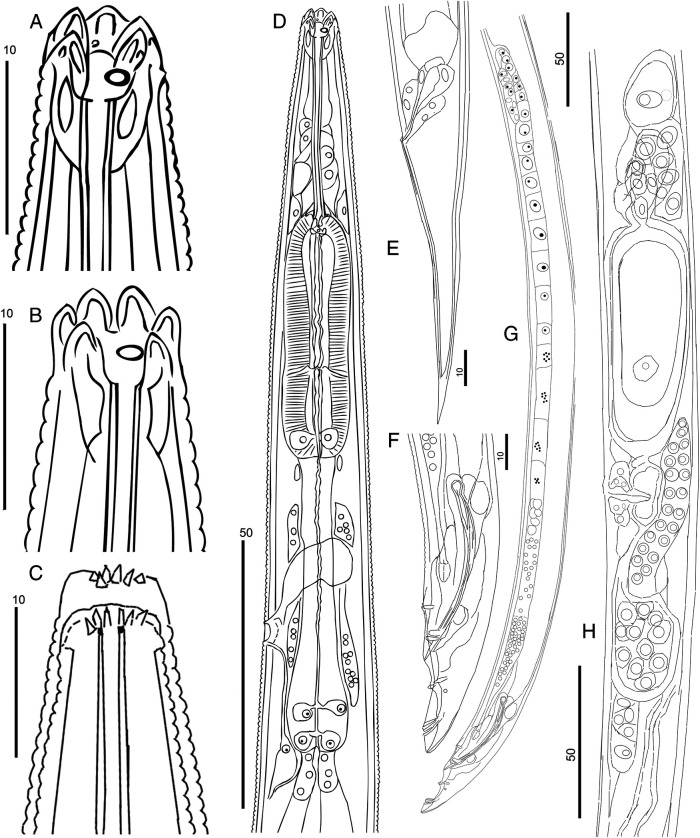
*Rhabditolaimus ulmi*. Morphology of adults. A – Male head; B – Female head; C – Head of molting juvenile (the molt of J4 into male); D – Male anterior part; E – Female tail; F – Male tail; G – Male genital system; H – Female genital system. Scale: 50 µm for D, G, H; 10 µm for rest.

**Figure 3:**
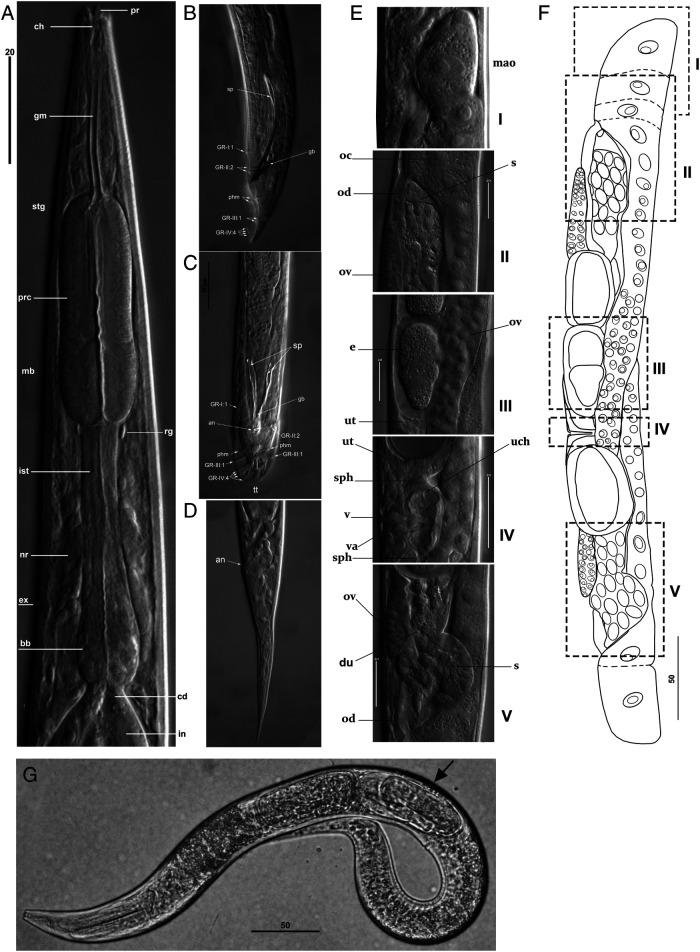
*Rhabditolaimus ulmi*. Morphology of adults. A – Female anterior part; B – Male tail, lateral view; C – Male tail, ventral view; D – Female tail, lateral view; E, F – Female genital system sections; G – Female with J2 in eggshell (arrow, ovoviviparity). The location of the fragments (E: I-V) is shown in the diagram (F). Fragments: I – Mature oocyte before reverse movement along the oviduct to uterus; II – anterior spermatheca and oviduct; III – anterior uterus with maturating egg; IV – vulva, vagina and uteral chamber bordered by two sphincters; V – posterior spermatheca and oviduct. an – anus; bb – basal bulb; cd – cardia; ch – cheilostom; du – duct between spermatheca and uterus; e – egg; ex – excretory pore; gb – gubernaculum; gm – gymnostom; GR-I:1…GR-IV:4 – groups of ribs on the bursa alae: I…IV – number of the group, P+1… 4 – number of papillae in a group; mao – mature oocyte; mb – median bulb of pharynx; nr – nerve ring; oc – oocyte; ov – ovary; phm – phasmid, pr – head probolae; prc – procorpus of pharynx; s – spermatheca; sg – stegostom; sp – spicules, sph – sphincter; tt – tail tip; uch – uteral chamber; ut – uterus; v – vulva; va – vagina. Scale: 50 µm for G; 20 µm for rest.

**Figure 4:**
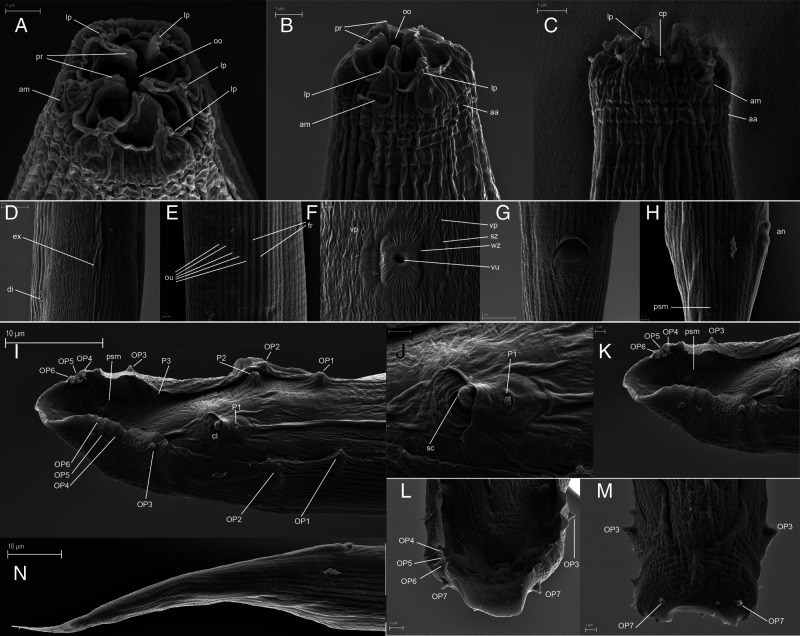
*Rhabditolaimus ulmi*. SEM. A, B – Female cephalic region. C – Male cephalic region; anterior annulus (aa), cephalic papilla (cp), amphid (am), lip papillae (lp), probolae (pr), oral opening (oo). D – Female excretory pore (ex) and deirid (di). E – Male lateral field at mid-body, lateral field ridges (fr), outer body ridges (ou). F – Vulva (vu) with vulval pore-like papilla (vp), wrinkled zone (wz) and smooth zone (sz). G, H – Female anus (an): G – Ventral view; H – Lateral view with a minute phasmid (psm). I – Male tail with caudal papillae, ventral view. P1 – unpaired precloacal papilla, OP1…OP7 – papillae on bursal alae, P2…P3 – inner papillae on ventral side of tail; cloacal opening (cl), pore-like phasmid (psm), ventral cultcular ridge (vr). J – Cloacal opening with spicule tips (sc) and P1 unpaired papilla. K – Tail end with OP3, group of OP4-OP6 papillae and phasmid (psm). L, M – OP7 papillae: L – Ventral view, M – Dorsal view. N – Female tail.

**Figure 5:**
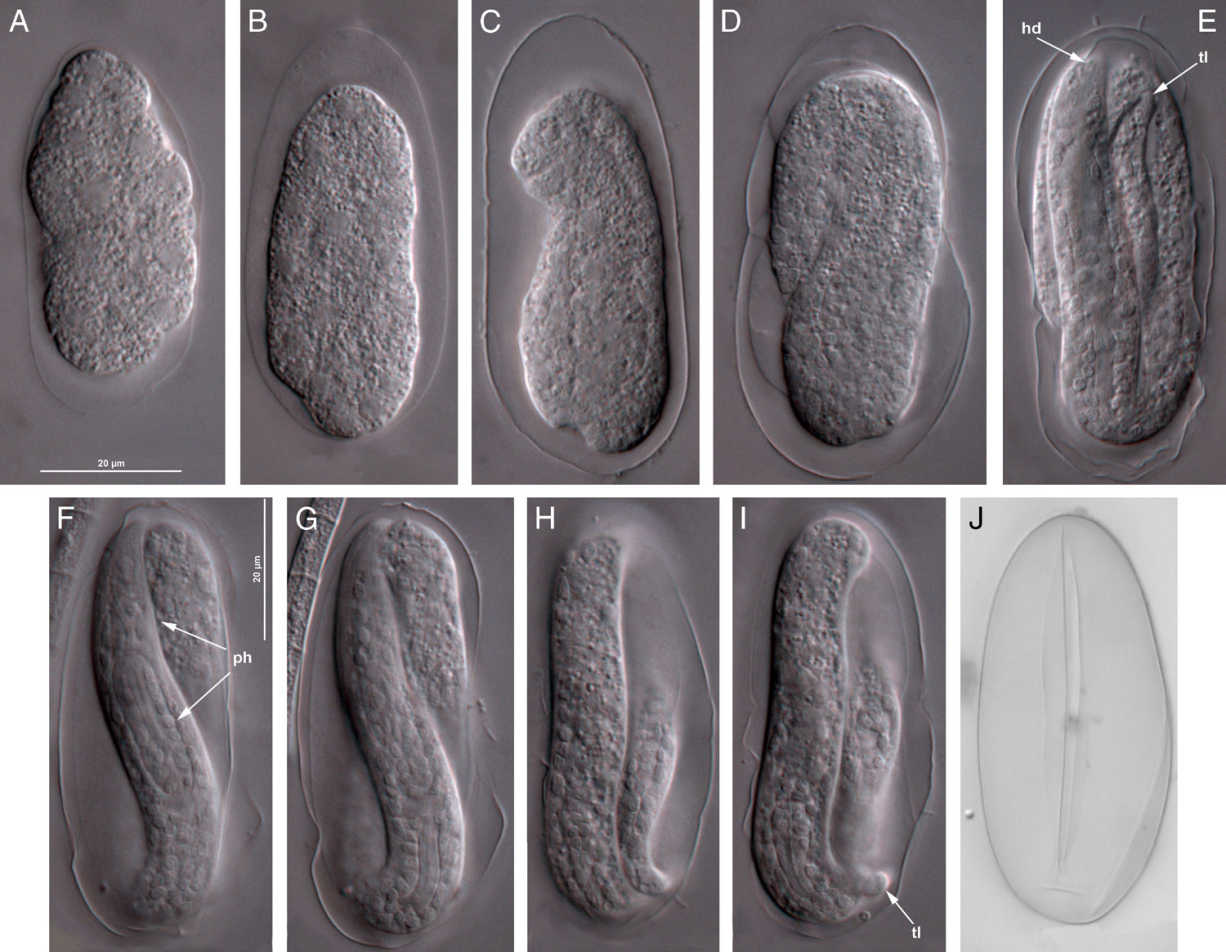
*Rhabditolaimus ulmi*. The development in egg. A to D – Embryo cleavage; E – Juvenile J1; F to I – Juvenile J2 at different focal levels; J – Egg shell with a longitudinal slit indicating the place from where the juvenile coming away. Scale 20 µm.

**Figure 6:**
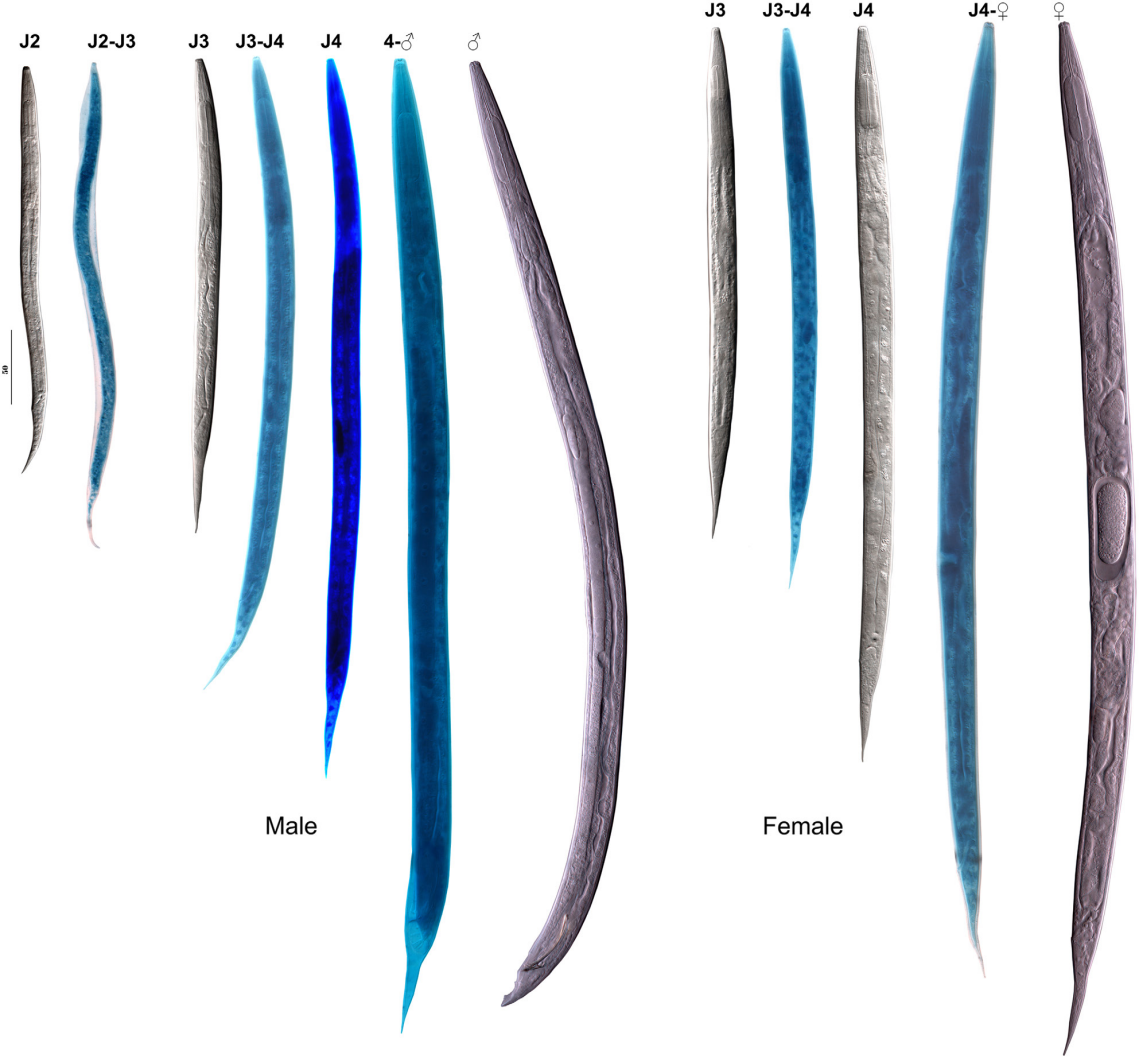
The juvenile development stages of males (left) and females (right). J1 .. J4 – juvenile stage numbers; pairs J2 to J3 .. J3-J4 – the molt of the junior stage to the senior one indicated by the stages numbers. hd – head; ph – pharynx; tl –tail. Scale 50 µm.

**Figure 7:**
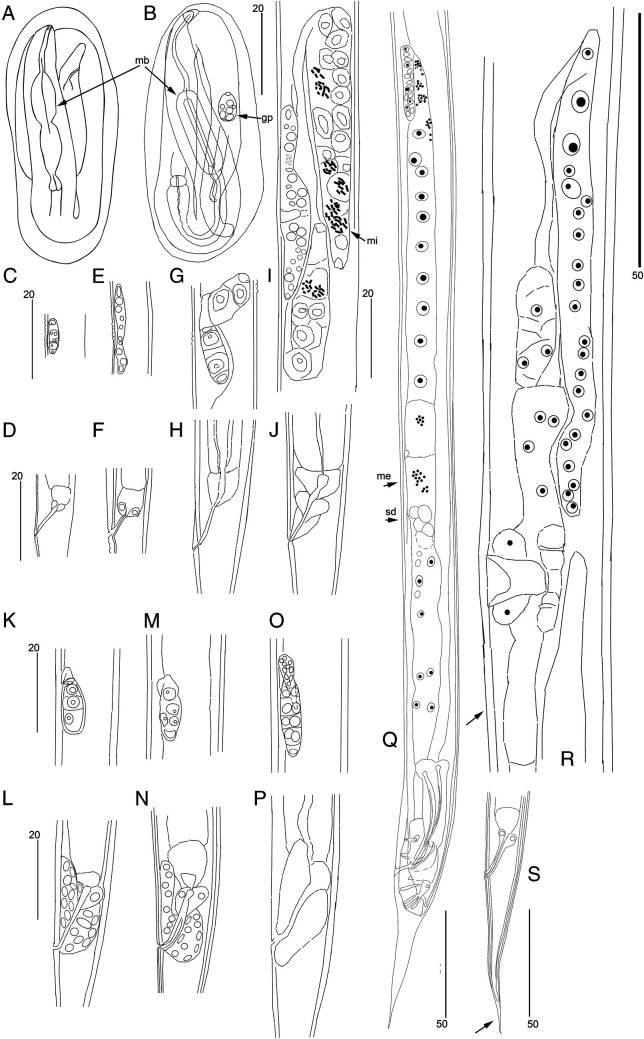
The development of juveniles of the propagative generation. A – Juvenile J1 in egg; B – Juvenile J2 in egg; genital primordia and anal regions of juveniles: C, D – Juvenile J2; E, F – Molt J2-J3; G, H – Juvenile J3 (female); I, J – Juvenile J4 (female); K, L – Juvenile J3 (male); M, N – Molt J3-J4 (male); O, P – Juvenile J4 (male, early molt phase); Q – Molt J4 – male; R, S – Molt J4 – female: R – Only anterior branch of genital primordium; S – Tail of molting female J4 (arrows – shed cuticule). gp – genital primordium; mb – median bulb of pharynx; me – meiosis; mi – mitosis; sd – spermatids. Scale: 50 µm for Q to S; 20 µm for rest.

**Figure 8:**
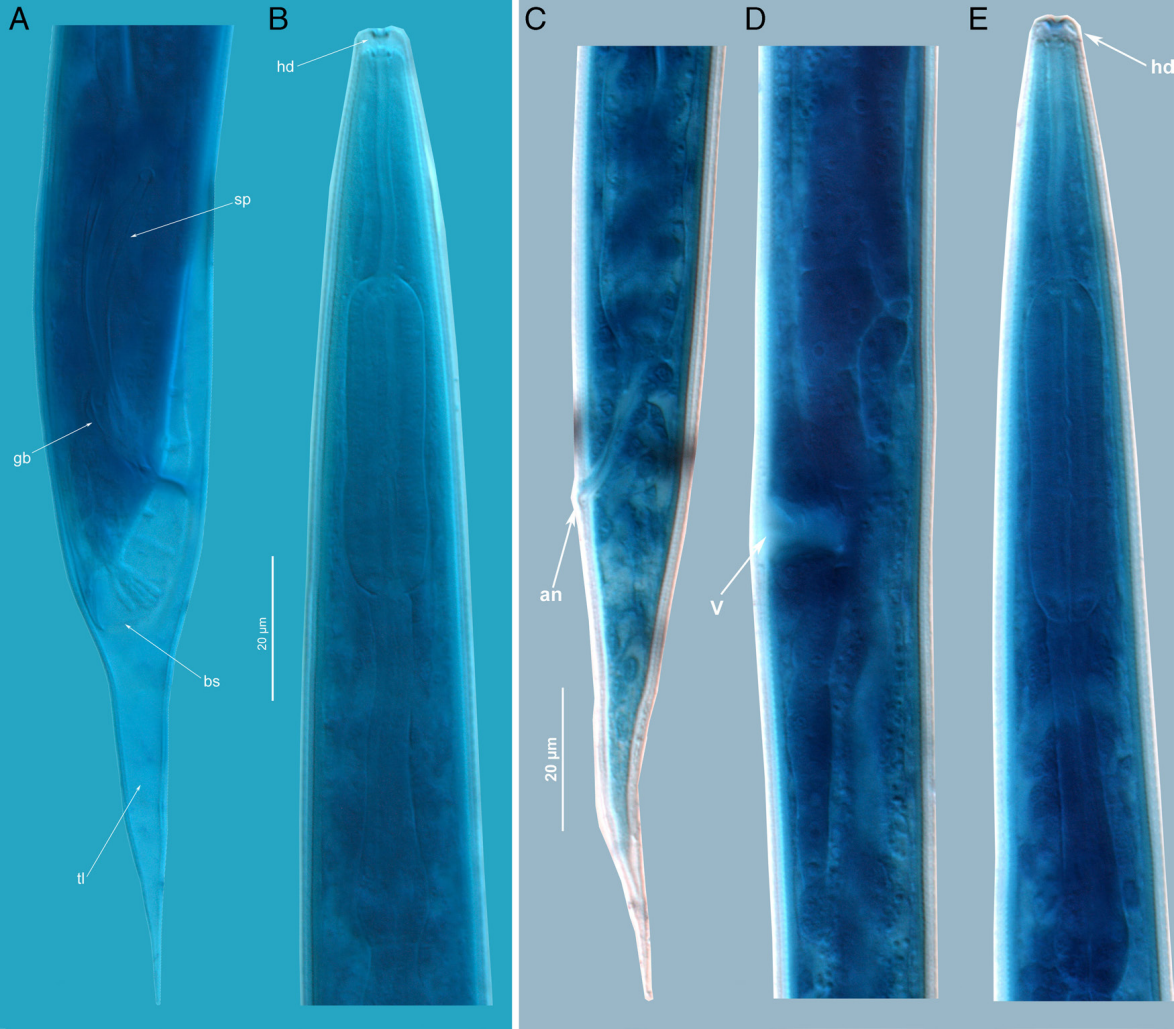
Molt of pre-adult juveniles J4 to adult male and female. Male tail (A) and its anterior part (B); Female: tail (C), Vulval region (D), Anterior part (E). an – anus; bs – bursa; gb – gubernaculum; hd – head with shed cuticle; sp – spicule; tl – tail. Scale: 20 µm.

**Figure 9:**
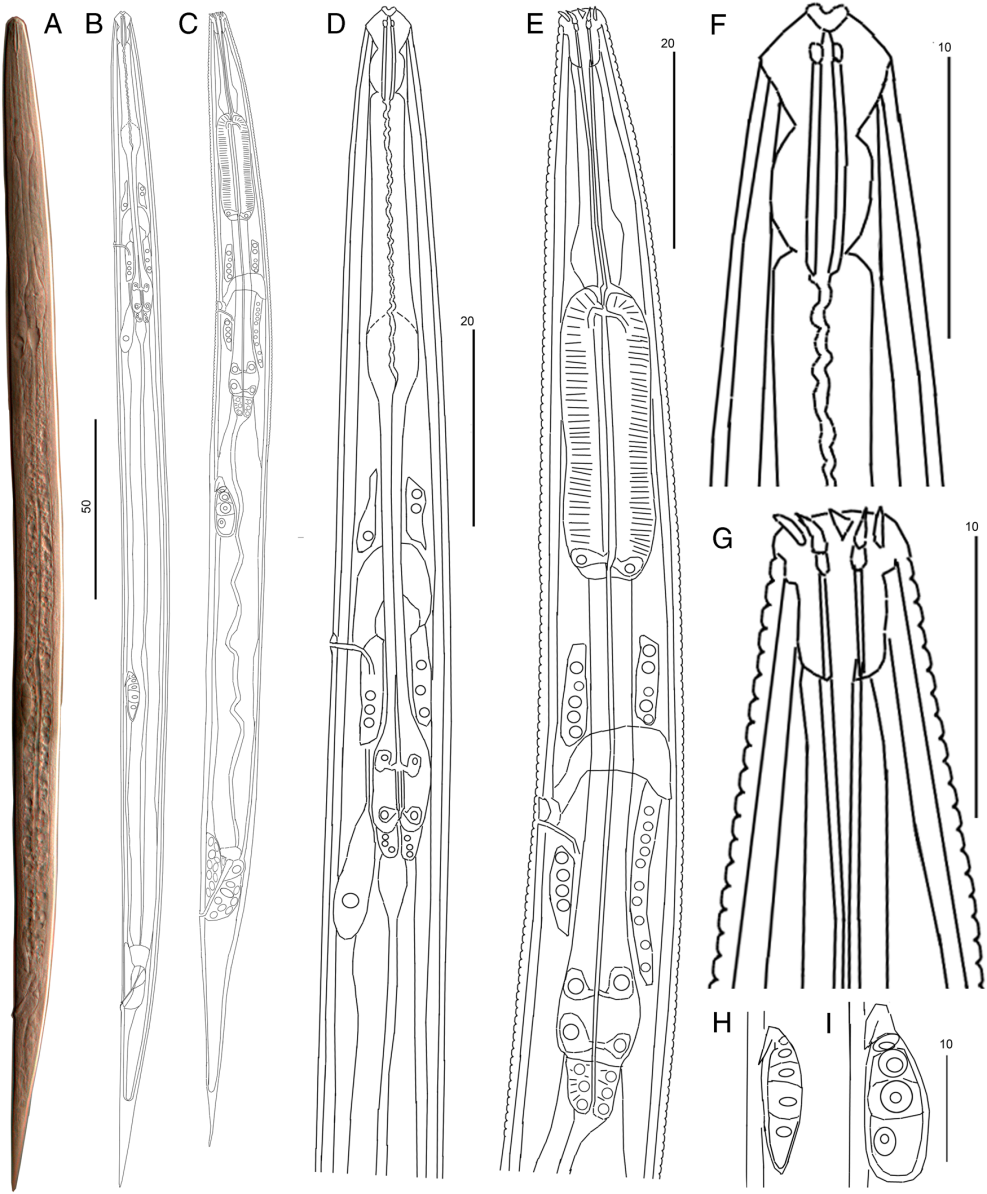
Dauer juveniles J3D of the transmissive generation and the juveniles J3 of the propagative generation (males). Total outline: A, B – Dauer juvenile; C – Juvenile J3 of propagative generation; anterior part and pharynx: D – Dauer, E – Juvenile J3; head: F – Dauer, G – Juvenile J3; Genital primordium (male): H – Dauer, I – Juvenile J3. Scale: 50 µm for A, B, C; 20 µm for D, E; 10 µm for F to I.

## Measurements

See Tables 1-3.

**Table 1.** Measurements (in µm) and ratios of *Rhabditolaimus ulmi* adults.

Character/sex	Adult male	Adult female
*n*	20	20
*L*	715 ± 148 (557-1007)	804.6 ± 139.2 (633-1012)
Body width	20.7 ± 5.2 (15.5-28.0)	27.0 ± 7.0 (19.0-36.0)
Stoma	35 ± 4 (29-40)	34.2 ± 3.6 (31-41)
Corpus (pro- and metacorpus)	41 ± 3 (34-47)	43 ± 4 (36-47)
Pharynx	130.6 ± 12.3 (112-149)	132.5 ± 11.7 (116-146)
Lip region width	6.4 ± 0.9 (5.5-8.0)	6.9 ± 1.0 (5.5-8.0)
Nerve ring from anterior end	101.7 ± 12.7 (84-127)	102.6 ± 8.6 (90-112)
Excretory pore from anterior	107.5 ± 13.1 (92-134)	111.6 ± 9.8 (97-127)
Tail	28 ± 7 (19-39)	83 ± 8 (68-92)
Anal body width	12.2 ± 1.7 (10-15)	12.4 ± 2.2 (10-16)
Genital tube length (GTL)	411 ± 100 (295-586)	403 ± 172.7 (204-760)
Genital tube width (GTW)	19 ± 8.8 (9.5-35.5)	27.6 ± 9.0 (15.5-39.0)
Spicule length along arc	45.9 ± 6 (34.5-53.0)	–
V %	–	56 ± 2 (55-60)
*a*	35.1 ± 4.4 (27.8-39.9)	30.6 ± 4.6 (26.1-38.0)
*b*	5.4 ± 0.7 (4.8-6.8)	6.1 ± 0.6 (5.3-6.9)
*c*	25.8 ± 4.0 (20.5-33.4)	9.7 ± 1.0 (8.5-11.5)
*c′*	2.3 ± 0.4 (1.7-3)	6.8 ± 1.0 (5.5-8.2)
Stoma/corpus	0.81 ± 0.02 (0.79-0.85)	0.82 ± 0.06 (0.74-0.87)
Stoma/pharynx	0.28 ± 0.01 (0.26-0.27)	0.27 ± 0.02 (0.25-0.29)
Corpus L/diam.	3.4 ± 0.3 (2.7-3.8)	3.2 ± 0.3 (2.7-3.6))
Corpus/pharynx	0.31 ± 0.03 (0.29-0.37)	0.32 ± 0.01 (0.30-0.36)
Pharynx anter./post.	0.60 ± 0.01 (0.59-0.61)	0.61 ± 0.04 (0.55-0.65)
GTL/GW	24.8 ± 10.8 (13.5-41.4)	14.7 ± 4.4 (10.8-23.3)
GTL/L, %	60 ± 10 (40-70)	50 ± 20 (30-90)

**Notes:** All values are given as mean ± s.d. (minimum-maximum); GTL/GW: ratio, genital tube length to its diameter; GTL/L: ratio, genital tube length to body length.

**Table 2.** Measurements (in µm) and ratios of *Rhabditolaimus ulmi* juveniles.

Character/stage	J2 in egg	J2 free	J3 male	J3 female	J4 male	J4 female	Dauer
*n*	5	10	7	11	7	10	10
*L*	190 ± 11.6 (176-200)	290.2 ± 31.4 (246-352)	358 ± 48 (292.6-427)	354.4 ± 38.6 (298-423)	475.6 ± 51.4 (373-558)	526.4 ± 66.8 (409-666)	338.8 ± 13.7 (326-354)
Body width	6.4 ± 0.6 (5.5-7.0)	7.8 ± 1.2 (5.5-10.5)	9.7 ± 1.5 (7.5-12.5)	9.6 ± 1 (7.5-12.0)	11.6 ± 1.1 (10-14)	12.6 ± 1.4 (9.5-16.5)	8.3 ± 0.3 (8.0-8.5)
Stoma	18 ± 3 (14-20)	23 ± 2 (21-26)	25 ± 2 (23-29)	25 ± 2 (23-29)	29 ± 2 (26-33)	30 ± 2 (25-35)	10 ± 1 (9-11)
Corpus (pro- & metacorpus)	22 ± 2 (20-24)	25 ± 2 (22-28)	27 ± 3 (21-31)	26 ± 3 (23-32)	33 ± 4 (28-41)	36 ± 5 (30-46)	33 ± 3 (30-37)
Pharynx	74.9 ± 12.9 (59-90)	91.7 ± 3.3 (86-97)	97 ± 7.4 (83-109)	92.6 ± 7.1 (87-111)	107.4 ± 7.8 (92-117)	119.4 ± 6.5 (106-132)	86.5 ± 3.9 (83-92)
Lip region width	1.9 ± 0.2 (1.5-2.0)	3.7 ± 0.3 (3.0-4.5)	3.9 ± 0.3 (3.5-4.5)	3.9 ± 0.7 (2.5-5.0)	4.5 ± 0.7 (3.5-6.0)	5.0 ± 0.8 (3.5-6.5)	4.2 ± 0.1 (4.0-5.5)
Nerve ring from anterior	–	66.2 ± 3.6 (61-76)	73.1 ± 5 (64-81)	70.7 ± 5.8 (62-80)	83 ± 9.2 (65-96)	88.6 ± 5.4 (81-99)	61.5 ± 1.7 (60-64)
Excretory pore	–	66.8 ± 4 (62-72)	79 ± 5 (73-86)	77 ± 5 (73-83)	84 ± 2 (82-86)	85 ± 2 (83-87)	65.4 ± 1.3 (64-671)
Tail	15 ± 3.9 (11-19)	46.3 ± 6.1 (35-60)	56.8 ± 9.2 (42-68)	55 ± 7.6 (38-65)	67.4 ± 6.6 (50-78)	71 ± 11 (41-88)	49.1 ± 0.5 (48-50)
Anal body width	3.5 ± 0.7 (2.5-4.0)	5.6 ± 0.8 (4.0-7.0)	7.3 ± 0.9 (5.5-8.5)	6.9 ± 0.9 (5.0-8.5)	8.9 ± 1.1 (7.5-11.5)	11 ± 1.6 (9.0-13.5)	5.3 ± 0.3 (5.0-6.0)
Genital primordium length (GPL)	–	7.7 ± 1.7 (5.5-10.5)	12.5 ± 3.6 (6.5-20.5)	13.4 ± 6.8 (6.0-29.0)	77.7 ± 48.3 (12.5-149.5)	100.8 ± 63 (11.0-170.5)	11.5 ± 1.5 (10.0-13.5)
Genital primordium width (GPW)	–	3.5 ± 0.8 (2.0-4.5)	4.1 ± 0.9 (2.5-5.5)	5.1 ± 1.5 (3.0-7.0)	6.4 ± 2.3 (3.5-12.0)	6.9 ± 1.3 (4.0-10.5)	3.8 ± 0.9 (2.6-4.7)
(V): GP center from anterior/ L, %	–	62 ± 4 (56-66)	54 ± 4 (49-57)	55 ± 4 (50-59)	51 ± 4 (46-55)	57 ± 4 (50-60)	57 ± 1 (56-59)
*a*	30.2 ± 3.5 (25.7-33.0)	37.5 ± 2.7 (33.4-43.1)	37.1 ± 4.3 (29.5-43.7)	37.3 ± 3.8 (27.9-43.0)	41.2 ± 2.1 (38.1-44.2)	41.7 ± 2.9 (36.4-44.5)	40.6 ± 1.4 (38.7-42.0)
*b*	2.6 ± 0.4 (2.2-3.2)	3.2 ± 0.4 (2.6-3.8)	3.7 ± 0.4 (2.9-4.2)	3.7 ± 0.2 (3.4-3.9)	4.4 ± 0.4 (3.6-4.9)	4.4 ± 0.4 (3.6-5.2)	3.9 ± 0.2 (3.7-4.1)
*c*	12.9 ± 2.6 (10.5-15.7)	6.3 ± 0.9 (5.3-8.3)	6.4 ± 0.7 (5.0-7.8)	6.5 ± 0.7 (5.7-7.9)	7.1 ± 0.7 (6.0-8.3)	7.6 ± 1.7 (6.2-13.5)	6.9 ± 0.3 (6.6-7.2)
*c′*	4.3 ± 0.8 (3.7-5.2)	8.3 ± 0.9 (6.2-9.6)	7.8 ± 0.9 (5.9-9.2)	8.0 ± 0.8 (6.1-9.2)	7.6 ± 1 (6.0-9.2)	6.7 ± 1.7 (3.0-8.7)	9.2 ± 0.4 (8.7-9.7)
Stoma/corpus	0.8 ± 0.2 (0.6-1.0)	0.9 ± 0.1 (0.8-1.1)	1 ± 0.1 (0.9-1.1)	0.9 ± 0.1 (0.8-1.1)	0.9 ± 0.1 (0.7-1.1)	0.8 ± 0.1 (0.6-1.1)	0.32 ± 0.03 (0.29-0.34)
Stoma/pharynx	0.23 ± 0.01 (0.22-0.24)	0.24 ± 0.02 (0.22-0.26)	0.25 ± 0.02 (0.23-0.27)	0.28 ± 0.02 (0.26-0.30)	0.27 ± 0.02 (0.24-0.29)	0.27 ± 0.02 (0.24-0.29)	0.12 ± 0.01 (0.11-0.12)
Corpus L/diam.	4.0 ± 0.4 (3.6-4.5)	3.6 ± 0.3 (3.2-4.4)	3.3 ± 0.4 (2.6-4.1)	3.5 ± 0.4 (2.7-4.2)	3.6 ± 0.4 (3.1-4.7)	3.6 ± 0.3 (3.0-4.1)	5.4 ± 0.5 (5.0-6.2)
Corpus/pharynx	0.30 ± 0.03 (0.26-0.34)	0.27 ± 0.02 (0.22-0.31)	0.27 ± 0.01 (0.25-0.29)	0.27 ± 0.01 (0.25-0.29)	0.3 ± 0.03 (0.2-0.3)	0.30 ± 0.04 (0.24-0.39)	0.37 ± 0.01 0.36-0.39)
Pharynx anter. part/pharynx L	0.53 ± 0.04 (0.48-0.57)	0.52 ± 0.03 (0.43-0.57)	0.54 ± 0.04 (0.48-0.57)	0.56 ± 0.04 (0.50-0.59)	0.55 ± 0.04 (0.50-0.59)	0.58 ± 0.04 (0.52-0.61)	0.48 ± 0.02 (0.47-0.51)
GPL/GPW	–	2.3 ± 0.5 (1.4-3.1)	3.2 ± 1.1 (1.7-6.2)	2.5 ± 0.7 (1.7-4.4)	11.4 ± 4.9 (2.6-17.2)	14.4 ± 8.9 (2.7-30.0)	3.1 ± 0.6 (2.6-3.9)
GPL/L, %	–	3 ± 1 (2-4)	4 ± 1 (2-7)	4 ± 2 (2-7)	16 ± 9 (3-27)	19 ± 12 (3-37)	4 ± 1 (3-5)

**Notes:** All values are given as mean ± s.d. (minimum-maximum); GP center from anterior/L: ratio, head to center of genital primordium/body length; GPL/GPW: ratio, genital primordium length to its diameter; GPL/L: ratio, genital primordium length/body length.

**Table 3.** Measurements (in µm) and ratios of molting juveniles of *Rhabditolaimus ulmi*.

Character/stage	J2-J3	J3-J4 male	J3-J4 female	J4-Adult male	J4-Adult female
*n*	2	5	5	5	2
*L*	283, 321	461 ± 22.9 (435-482)	445.5 ± 67.3 (378-525)	582 ± 81 (472-669)	643, 646
Body width	6.0, 7.0	12.8 ± 0.9 (11.5-14.0)	12.0 ± 1.6 (10.5-14.5)	14.2 ± 1.6 (12-16)	15.5, 16.0
Stoma	21, 22	31 ± 3 (26-34)	29 ± 3 (24-32)	33 ± 3 (29-37)	33, 36
Corpus (pro- and metacorpus)	25, 26	34 ± 2 (31-35)	30 ± 5 (23-37)	39 ± 4 (35-45)	44, 48
Pharynx	88, 94	122 ± 10 (109-130)	118 ± 12 (103-131)	129 ± 9 (118-141)	139, 145
Lip region width	3.5, 4.0	5.1 ± 0.8 (4.0-6.0)	5 ± 1 (4.5-6.5)	6.2 ± 1.2 (5.0-7.5)	6.5, 7.0
Nerve ring from anterior end	65, 66	82 ± 5 (77-90)	81 ± 9 (69-93)	95 ± 6 (88-101)	97, 101
Tail	47, 53	64 ± 4 (59-69)	64 ± 6 (59-73)	71 ± 4 (67-75)	74, 76
Anal body width	4.5, 5.0	9.4 ± 1.1 (8.0-11.0)	7.6 ± 0.6 (7.0-8.5)	12.4 ± 2.3 (9.5-15.0)	10.0, 11.0
Genital primordium length (GPL)	7, 10	31.9 ± 13.2 (13-49)	21.2 ± 17.6 (9-52)	226.6 ± 111.2 (95-357)	145, 262
Genital primordium width (GPW)	2.0, 3.0	6.6 ± 0.9 (6.0-8.0)	6.1 ± 1.1 (5.5-8.0)	10.7 ± 4.6 (5.5-15.5)	9.5, 12.0
Spicule length along arc	–	–	–	34.5-42.0	–
(V): GP center from anterior/L, %	47, 55	55 ± 4 (49-58)	55 ± 3 (49-57)	88 ± 6 (79-93)	50, 59
*a*	40.0, 54.3	36.1 ± 1.2 (34.3-37.4)	37.5 ± 7 (26.4-45.5)	40.9 ± 2.2 (38.5-43.4)	40.0, 41.8
*b*	3.0, 3.6	3.8 ± 0.1 (3.6-3.9)	3.7 ± 0.2 (3.4-4)	4.5 ± 0. 5 (3.8-4.9)	4.5, 4.6
*c*	6.0, 6.1	7.0 ± 0.5 (6.6-7.8)	7.0 ± 0.6 (6.2-7.6)	8.2 ± 0.8 (7.0-8.9)	8.5, 8.7
*c′*	10.3, 11.7	6.9 ± 1 (5.6-8.4)	8.3 ± 0.8 (7.2-9.4)	5.8 ± 0.8 (4.9-7)	7.2, 7.6
Stoma/corpus	0.8, 1.0	0.9 ± 0.1 (0.8-1.0)	1.0 ± 0.2 (0.9-1.2)	0.8 ± 0.1 (0.7-0.9)	0.7, 0.8
Stoma/pharynx	0.23, 0.25	0.25 ± 0.01 (0.24-0.26)	0.24 ± 0.01 (0.23-0.25)	0.25 ± 0.01 (0.24-0.26)	0.24, 0.25
Corpus L/diam.	4.0, 4.5	3.7 ± 0.2 (3.5-4.0)	4.2 ± 0.6 (3.7-5.3)	3.8 ± 0.6 (2.9-4.3)	4.0, 5.2
Corpus/pharynx	0.27, 0.3	0.28 ± 0.01 (0.26-0.30)	0.25 ± 0.03 (0.21-0.28)	0.31 ± 0.02 (0.28-0.34)	0.30, 0.35
Pharynx anter. part/ pharynx L.	0.51, 0.52	0.53 ± 0.01 (0.52-0.54)	0.50 ± 0.02 (0.46-0.53)	0.56 ± 0.02 (0.54-0.59)	0.55, 0.58
GPL/GPW	3.2, 3.4	4.8 ± 1.6 (2.1-6.1)	3.6 ± 3.3 (1.7-9.4)	20.8 ± 3.5 (17.0-24.7)	15.1, 21.7
GPL/L, %	2, 3	7 ± 3 (3 - 11)	5 ± 3 (2-12)	37 ± 14 (20-55)	20, 40

**Notes**: All values are given as mean ± s.d. (minimum-maximum).

### Adults, propagative generation

#### General morphology

Body almost straight, with weak annulation; width of 10 cuticle annuli at mid-body 7.5 µm. Lateral field starts at level of a telostom bulb continuing to the hyaline zone of a tail tip in females and to cloacal opening in males. The field marked by four incisures: its two prominent lateral ridges separated by a hollow where width equals the width of a ridge. On the ventral and dorsal side of the lateral field bordered by 3 to 5 lower ridges, crossed by transverse annulation, the maximum number of these ridges outside the lateral field is five at mid-body; decreasing to three ridges at body extremities. Outside the ridges the cuticle annuli are crossed by longitudinal lines; thus each annulus consists of rectangles where the long sides are parallel to the axis of the body. The prominent dorsal and ventral ridges of the cuticle beginning at the level of combined procorpus and first pharyngeal bulb; the dorsal ridge ends near the hyaline zone of tail tip in female and at the level of the cloaca opening in males; the ventral ridge ends in front of cloacal opening in males and before the anterior lip of anus in females. Cephalic region continuous, low, its sides angular in lateral view and hexagonal radially symmetrical in anterior view; devoid of annulation. With SEM, the cephalic area clearly separated by the first body annulus. Six triangular identical probolae point to the center of the six-lobed oral opening, forming a conical cupola. At the anterior margin of each probola is a tubular inner lip papilla with a central hole. Cephalic papillae absent in female but four tubular cephalic papillae are distinct in male in the middle of two subdorsal and two subventral probolae. Amphids oval, located behind the lateral probolae immediately behind the level of the posterior folds of the oral opening between cephalic lobes; the long amphid axis transversal, it equals the width of the base of probolae, its longitudinal axis is half as wide. Cephalic framework moderate, its basal plate at the probolae bases. Cheilostom cup-shaped, consisting of adradial cuticular plates, devoid of denticles; its front edge is connected to the six-looped oral opening of the body surface cuticle. Tubular base of cheilostom overlaps the anterior margin of triangular flexible gymnostom, encircled by muscular collar where length equals head width. Gymnostom 8-12 times longer than cheilostom, ending in stegostom in the anterior part of a first bulb. Stegostom is anisomorphic with distinct dorsal anterior bulge; the posterior part of stegostom encircled by a cuticular ring with two distinct lateral foramens. The first bulb muscular, its width 3 to 4 times of its length; it consists of two parts weakly separated by interruption of the triangular inner canal lining: its anterior part is a procorpus and its one-third posterior part is a homolog of metacorpus (median bulb of the Rhabditida); the metacorpus part of the muscular bulb includes a pair of unicellular glands with ducts opening into pharyngeal lumen. Isthmus is muscular gradually continuing to the pyriform second (posterior) bulb; canal within isthmus triangular thin walled. Cuticle ring encircled isthmus at the base of median bulb. Nerve ring in the middle of the isthmus, with ganglia anterior and posterior to it. Excretory pore at the posterior edge of the nerve ring. Deirid pore-like, its diameter is half of that of excretory pore, situated on the central hollow of lateral field between the field ridges, 3-5 annuli posterior to excretory pore. Hemizonid three annuli wide immediately anterior to excretory pore. Second bulb without grinder or valve; two pairs of unicellular glands are distinct in the basal part of bulb. Cardium distinct, at 1/3 bulb length, dividing pharynx and mid-intestine, its length twice its diameter; consisting of three rows of three cells. Mid-intestine cellular, wide, its wall consisting of one layer of large granular cells, its inner lumen 1/3 intestine diam. with distinct hyaline layer. Paired phasmids small pore-like with diameter equal to that of deirid, located one anal diameter posterior to anus, in the central hollow between the field ridges.

#### Male

Body moderately curved to ventral side. Genital system at right side, 2/3 of mid-intestine length. Testis anteriorly reflexed to branch of 1 to 2 body diam. Spermatocytes in multiple rows in reflexed branch, posteriorly arranged in one row of large elongated cells; spermatids as 1 to 3 quartets in the middle zone of gonad; followed by the tubular vas deferens, filled with secretory granules and small spherical sperm; the gonad opening into cloaca sac ventral, at 20% of spicule length, while the mid-intestine opens via small valve into cloaca dorsally between heads of spicules; three large rectal glands at sides of valve. Spicules paired and equal, very long, slightly curved, needle-like, heading small, needle-lug-shaped. Gubernaculum Y-shaped. Bursa alae enveloping tail tip, starting anteriorly at the level of last third of spicules. Hyaline tail tip narrowly conical, surrounded by bursa. Cloacal opening is a transverse slit anteriorly arch-shaped. Unpaired papilla-like papilla P1 two cuticle rings anteriorly to cloaca opening. It is transversely elongated, twin, with two connected tubular pores.

It is reasonable to divide the rest of the male tail papillae into three groups according to their positions in structure rows (i) outer paired papillae on the fold of bursal alae (7 pairs of papillae: OP1…OP7); (ii) inner paired papillae on the ventral tail surface (two pairs, their numeration continues that of unpaired papilla P1, abdominal: P2...P3); a pair of paired pore-like papillae, presumably homologous to phasmids of the female, locating on the tail surface at the inner base of lateral wings at the level of upper edge of triad OP4, OP5, OP6. The papilla-like papillae located on folds of bursal alae form bursal ribs. A pair of OP1 one anal diameter anteriorly to the cloacal opening mark start points of the bursal alae. Paired OP2 at the level of half the distance from OP1 to the cloacal opening. Paired OP3 at the level of mid- tail. Pairs OP4, OP5 and OP6 form a group that are close to the posterior edge of the bursa. Paired OP7 at the dorsal side of the bursal alae at a distance of two cuticle annuli from the bursal terminus, immediately behind the grouping of OP4, OP5 and OP6. The paired OP7 devoid of ribs; they form the posterior horns of the shovel-shaped end of the bursa, without ribs. Inner (abdominal) tail papillae. P2 at OP2 level at the base of the bursal alae; P3 anteriorly to the OP3 locating from the latter at the distance equal to that of OP3-OP4, i.e. three cuticular rings.

#### Female

Genital system paired, anterior branch at right side and posterior to left side of intestine. Ovaries reflexed (antidromous) with multiple rows of oocytes in both terminal endings. The mature oocyte enlarged at the loop of ovary flexure. Spermatheca filled with large cytoplasmic sperm, oval, its length twice of its diam. Spermatheca is the dorsally branching blind appendix of uterus that joins with ovary via narrow duct. Mature large oocyte passes the oviduct to uterus; and thus pushes the spermatheca; the latter injects sperm into uterus where the oocyte insemination occurs. Uterus paired, oval, anteriorly joined with oviduct ventrally and spermatheca dorsally; posteriorly the uterus connected with small oval uteral chamber bordered laterally with two pairs of dense thick-walled cells; the uteral chamber separated with two sphincters from both uteri: anteriorly from anterior uterus and posteriorly from posterior uterus. The chamber opens into cuticle vagina, perpendicular to ventral body surface; vagina opens into transversal vulva surrounded by valve, pyriform in lateral view. The valve surrounded with massive vulval muscles. The vulval hole is rounded, surrounded by dense cuticle folds that form a wrinkled oval-shaped vulval zone, the length of this zone is one and a half times more than its width. On the outside, the wrinkled zone is surrounded by a smooth round cuticle area, the width of the smooth zone equals to the width of the wrinkled zone. Outside of the smooth zone, the cuticle is annulated with a rectangular pattern typical to the rest of body surface. In front of the vulva at the edge of the smooth and annulated zones, two pore-like papillae are located at the level of front edge of the wrinkle zone. Their arrangement may be asymmetrical – anterior vulval papilla may be situated by the width of one cuticle ring at front of the other one.

Rectum 1.2 to 1.5 anal body diam., consisting of capillary posterior part and broad pyriform anterior part with wide lumen, bordered by three large rectal glands. Tail conical, 4 to 5 times its diam., gradually tapering to hyaline narrowly conical tail tip with hyaline zone of 1/6 of tail length. The anterior lip of female anus rounded, arch-shaped with marked cuticular border. Posterior anal lip is bulged, smooth in front and posteriorly annulated; annuli are crossed by longitudinal incisures breaking the cuticle rings into longitudinal elongated rectangles, just behind the posterior anal lip seven rows of rectangles form a V-shaped figure, posteriorly at a distance of one anal body width from anus the number of rows decreases to five and they are parallel.

#### Eggs

Egg is large, its length 58 to 65 µm; 2.5 times of its diam. Eggs are laid by a female at different stages of cleavage: from single cell stage up to the second stage juvenile after the first molt inside eggshell; in latter case it is the ovoviviparity. Eggs are usually laid in chains of 2 to 5 units. Eggshell is smooth but after a juvenile breaks the shell its surface is marked with longitudinal slit.

### Juvenile stages grown in the laboratory culture

#### Development in an egg

Propagative females laid eggs at different stages: from a blastula to an egg containing a completely developed J1 or J2; the latter case is ovoviviparity. The J1 juvenile inside the eggshell had a distinct pharynx with a small medial bulb without a valve, a posterior bulb and cardia. The stoma is indistinguishable. The second stage juvenile J2 inside the eggshell had an external shed cuticle and a massive long bulbous pharynx corpus (first bulb) with a triangular cuticular lumen, which is similar to that structure in adult females of the propagative generation. The genital primordium of at least six cells is visible. At this stage, the J2 juvenile was hatching from the eggshell, breaking through a longitudinal slit by its head end. The J2 juvenile within egg rolled up in three curves. After leaving the egg the J2 straightened, its body length elongated.

### Stages of development after leaving the eggshell

#### J2 juvenile, where sex is not recognizable

Except for genital structures, the J2’s general morphology is similar to that of the adult female. Small elongated genital primordium in the middle of the mid-intestine part of the body. Primordium consists of eight cells: four small somatic cells in the central part, each of two primordium extremities with one large germinal cell and one small apical cell.

#### J3 male

Except for genital structures, the J3 male’s general morphology is similar to that of the adult male. Small drop-like genital primordium in the middle of the mid-intestine part of the body. Four large germinal cells with an apical cell in posterior pole; in anterior part of primordium a small hook-like part with somatic nuclei reflexed posteriorly. This part in later stages will grow to cloaca primordium. Cloaca primordium present, encircling rectum, with its ventral side narrow and elongated anteriorly and dorsal side widely expanded.

#### J3 female

Except for genital structures, the J3 female’s general morphology is similar to that of the adult female. Vulval primordium absent. Genital primordium is a small Z-shaped to horseshoe shaped structure with central somatic zone and two flexed branches, each of them with two large germinal cells and small apical cell, situated in the middle of mid-intestine section of the body.

#### J4 male

Except for genital structures, the J4 male’s general morphology is similar to that of the adult male. Genital primordium horseshoe-like, in the middle of the mid-intestine part of the body, its length 5 to 7 times of its diam. Main part of primordium with 10 to 15 large germinal cells and with a small apical cell on posterior pole. Anteriorly primordium has a hook-like reflexed part with somatic nuclei which grows during this stage in the direction of cloaca primordium, finally joining with the latter. Cloaca primordium encircling the expanded rectum canal; its ventral side narrow and elongated anteriorly and dorsal side widely expanded. Primordium has very dense structure filled with numerous somatic nuclei.

#### J4 female

General morphology is the same as in adult female with smaller sizes of organs. The only difference is the genital structures. Genital primordium 4 to 5 body diam. long; it consists of two equal reflexed branches with central part with vulval primordium attached to the ventral body wall in the middle of the mid-intestine section of the body. Anterior branch at right side and posterior branch at left side of the mid-intestine. Vulval primordium 1/5 body diam., massive, containing 30 to 40 somatic nuclei, with the central transverse lens-like invagination under cuticle. Genital primordium attached to dorsal surface of vulval primordium. Reflexed part of each branch of genital primordium consists of ca 10 large germinal cells and the apical cell, almost reaching the vulval primordium invagination. Non-reflexed part of genital primordium tubular, consisting of somatic cells.

#### Molting individuals

The molting individuals (between stages JN and JN + 1) are characterized by the presence of shed cuticle and the genital primordia with structures which are intermediate between those typical for the non-molting specimens of JN and JN + 1. Two preadult molting phases are described below.

#### J4-Adult male molting juveniles

Body in shed molting cuticle, separated from body at extremities; it is remarkable that the shed elongated conical tail cuticle envelops the rounded male tail with bursa. All structures as in adult male, including caudal bursa and its papillae, except for the weak and very transparent spicules in cloaca canals.

#### J4-Adult female molting juveniles

Body in shed molting cuticle, separated from body at extremities. All structures as in adult female, except genital tube. Vulval-vaginal primordium is a transparent ventral wide invagination under cuticle which dorsal bottom is shaped as provisory uteral chamber. Two branches of genital primordium equal in size, fused with vulval-vaginal primordium attaching to the provisory uteral chamber. Each branch is divided into sections of future uterus, spermatheca, oviduct and the reflexed provisory ovary of 15 to 20 germinal cells reaching the vaginal primordium from dorsal side. Anterior genital primordium branch at right side and posterior at left side of mid-intestine. Tail conical as in female, anus and rectum well developed, three rectal glands visible.

All nematodes described above were initially collected from the bark and wood and then multiplied in agar cultures with the fungus *Botryotinia fuckeliana*. Additionally, transmissive dauer juveniles (DJ3) were collected from the undersides of the elytra of *Scolytus multistriatus*.

#### Dauer (the juvenile DJ3 of entomophilic transmissive generation)

(Fig. 9 and Table 1).

Body straight. Annulation weak, four incisures in lateral field. Deirid indistinct. Cephalic region continuous, high, conical, probolae absent, lips amalgamated; with labilal disc. Cephalic framework weak hyaline-like. Stoma slit-like, thick-walled, short, 5 to 8 its widths. Pharynx narrow. Procorpus and metacorpus joined in elongated pyriform corpus, but with distinct border between long cylindrical procorpus and compact ellipsoid metacorpus, the latter with distinct inner triangular canal and devoid of cuticle valve. Isthmus narrow, with capillary canal, posterior bulb elongated ovoid; with two paired gland ducts opening into the canal. Cardium of ¼ bulb length, collar-like, separating pharynx from mid-intestine. Nerve ring at mid-isthmus; excretory pore at its posterior border; hemizonid 3 annuli wide just anterior to excretory pore. Genital primordium ventral, at ½ distance of mid-intestine; in female juveniles U-shaped with terminal germinal zones each with two large cells and central somatic part with ca 10 small nuclei; in male juveniles primordium drop-like with anterior hook-like somatic part and the germinal part of 4 large cells directed posteriorly. Rectum 1.5 times anal body diam., posterior part thickened, anus distinct. Tail conical, straight, 9 to 10 times anal body diam; terminal hyaline zone equal to rectum length. Phasmid small, at anterior third of tail. Male dauer juvenile differs from female dauer juvenile in presence of cloaca primordium around the thickened posterior half of rectum; its diam. equals to ¾ of anal body diam. According to 300 to 360 µm body size and genital primordium with 4 germinal cells the dauer juveniles correspond to the J3 juveniles of the propagative generation and therefore identified as the DJ3 stage of transmissive generation. After 3 to 6 hr in water or on PA media the DJ3 molted to DJ4 and then to adult nematodes with well-developed stoma and pharynx as described above for males and females.

#### Diagnosis

Cephalic region and stoma hexagonal; female with six lip sensilla and two amphids, male with additional four cephalic sensilla. Didelphic females and males with peloderan bursa; papilla formula: three preanal pairs, one unpaired precloacal; seven postanal pairs (five subventral papilla-like, one subdorsal papilla-like; one ventral pore-like glandular). Corpus massive, 3 to 4 diam. long; stoma 0.8 times corpus length. Lateral field 2 ridges and hollow (4 incisures), additional 3 to 5 ridges from each side of lateral filed crossed by cuticle annuli. Commensals of bark beetles *Scolytus* spp.

#### Relationships

*Rhabditolaimus ulmi* similar to *R. zamithi*, *R. robiniae* and *R. walkeri* in combination of didelphic female genital system and peloderan bursa. *R. ulmi* differs by having longer spicules: 34 to 53 µm vs 25 to 28 µm in *R. zamithi*, 30 to 35 µm in *R. robiniae*, and 24 to 29 (27) µm in *R. walkeri. R. ulmi* has the highest corpus ratio (length to diam.): 3 to 4 vs 3 or less in *R. zamithi*, *R. robiniae*, and *R. walkeri.* Value *c′* 5.7 to 7.4 (6.6) is the highest value for females in *R. ulmi* vs 4.9 to 5.0 in *R. robiniae* and 3.4 to 4.9 (4.4) in *R. walkeri.* Additionally, the male caudal papillae of *R. ulmi* pattern (3 + 1 + 7) is unique in the presence of unpaired pre-cloacal papilla and the subventral pore-like papillae pair which are possible phasmids homologs. *R. zamithi*, *R. robiniae* and *R. walkeri* have the pattern of 3 preanal + 6 postanal papilla pairs.

Key to juvenile stages and adults of *Rhabditolaimus ulmi*:

- Copulatory structures functional, body length equal to or longer than 550 μm. ...............2

- Copulatory structures absent, mean body length shorter than 550 μm. ....................3

- Tail with paired cuticular spicules, caudal bursa present, genital system in one branch from cloaca along most of intestine body section. ..............................Adult male

- Vulva as transverse slit-like opening in the middle of intestine body section present, genital system of two opposite branches outgoing from vulva. .........................Adult female

- Juveniles in beetle larvae, pupae and imagoes, mostly in buccal cavity and ovipositor cavity of insects, head high, conical with hyaline framework, probolae not developed, stoma and pharynx reduced. .......................dauer juvenile

- Juveniles in wood and bark, head low, truncate, well developed six probolae, stoma and pharynx. ........................................4

- Body shorter than 350 µm, genital primordium very small cylindroid, consisting of 8 cells: small apical on poles, 2 large germinal cells divided by 4 small somatic cells in the center. ........J2

- Mean body length more than 350 µm; genital primordium larger with 4 or more large germinal cells and 10 or more somatic cells, its shape mostly reflexed one or two times, or in a form of horshoe ..........................................5

- Cloaca primordium around rectum present. .........................6 (male juveniles)

- Cloaca primordium absent 7....(female juveniles)

- In genital primordium 4 germinal cells, its length three its diam. ...................J3 male

- In genital primordium 10 and more germinal cells, its mean length 10 or more of its diam. ....................................J4 male

- Vulva primordium present, transparent lens-like under cuticle in the middle of intestinal part of body, genital tube in a form of two equal reflexed branches outgoing from the vulval primordium and occupying one third of intestine body section. .........................J4 female

- Vulva primordium absent, genital primordium less than 1/10 of the intestine part of the body. ...................................J3 female

#### Locality of studied material

Park of St Petersburg State Forest Technical University (59.991923°N, 30.342697°E), St Petersburg, Russia.

#### Vector and plant host

Cultures on *B. fuckeliana*-PA medium were started from individuals isolated from the bark and soft wood (1 cm deep) obtained from a dying elm, *Ulmus glabra* (Ulmaceae) showing symptoms of Dutch elm disease, i.e., wilting, dark-colored ring in cross-section of wilted branches, trunk with galleries of larvae, pupae and imagoes of *S. multistriatus* (Curculionidae: Scolytinae). Additional cultures on *B. fuckeliana*-PA medium were started from dauers collected under elytra of imagoes of *S. multistriatus.*

#### Nematode collection

Collection slides were deposited at the State Collection of the Zoological Institute RAS (20 slides) and the University of California, Davis Nematode Collection, CA, USA (10 slides).

#### Genus diagnosis

*Rhabditolaimus* Fuchs, 1914.

(Generic synonymy according to Susoy and Herrmann, 2012).

*Diagnosis (emended):* Diplogastridae. Lateral field of two ridges separated by hollow (four incisures); outside the field the additional less prominent ridges crossed annules. Cephalic region and stoma hexagonal; female with six lip sensilla and two amphids, male with additional four cephalic sensilla. Cheilostom with inner cuticular plates, without denticles; its outer wall overlaps anterior end of gymnostom. Gymnostom flexible, very long, 10 times of its width and longer, ratio: gymnostom to corpus 0.4 to 1.3; a ratio stoma (cheilostom and gymnostom) to pharynx 0.2 to 0.4. Stegostom in the anterior part of corpus, asymmetric with anterior dorsal bulge and with posterior inclined subventral plates without denticles; it is encircled posteriorly with cuticular ring with two lateral foramens. Corpus strong cylindrical muscular bulb, 2 to 5 widths long, combining procorpus and metacorpus which borders are marked by interruption of the lumen triangular lining. Excretory-secretory pore at second bulb which devoid of grinder valve, or the pore shifted to base of pharynx. Hemizonid just anterior to excretory pore. Deirid a small pore at nerve ring or shifted to the base of pharynx. Female genital system didelphic with central vulva, or monoprodelphic with vulva shifted posteriorly (*V* = 55 or more). In females two small pore-like phasmids in the anterior part of the long conical tail. Male with well-developed peloderan or leptoderan narrow bursa, in type-species and bursa enveloping tail but with a mucronate 2 to 10 µm spike outside the bursal flap. Generally three preanal and six postanal caudal papilla pairs (sometimes 2 preanal and 5, 7 or 8 postanal pairs); the postanal papilla located at bursa alae and ventrally, a group of closely situated 3 papilla pairs (ribs) at the posterior margins of alae; one of postanal papilla is subdorsal. In some species unpaired pre-cloacal papilla detected and one of the paired postanal papilla may be pore-like: they are the ‘glandular papillae’ which may be homologs of phasmids. Male spicule narrow, widely varied in size from 17 to 600 µm. Gubernaculum present, with complicated species-specific shape. Saprophages and phoronts of bark-beetles with the specific dauer juveniles of transmissive generation. Some species associated with soil and decaying matter.

### Type species: *Rhabditolaimus leuckarti* Fuchs, 1914

#### List of the *Rhabditolaimus* species

Here is used the list of species names accepted as valid in Susoy and Herrmann (2012) with their synonyms.

#### *Rhabditolaimus leuckarti* Fuchs, 1914 – the type species

*R. anoplophorae* (Kanzaki and Futai, 2004) Susoy and Herrmann (2012)

*R. carolinensis* (Massey, 1967) Susoy and Herrmann (2012)

*R. curzii* (Goodey, 1935) Susoy and Herrmann (2012)

*R. dendrophilus* (Kinn, 1984) Susoy and Herrmann (2012)

*R. erectus* (Massey, 1960) Susoy and Herrmann (2012)

*R. goodeyi* (Rühm, 1959) Susoy and Herrmann (2012)

*R. inevectus* (Poinar, Jackson, Bell and Wahid, 2003) Susoy and Herrmann (2012)

*R. kishtwarensis* (Hussain, Tahseen, Khan and Jairajpuri, 2004) Susoy and Herrmann (2012)

*R. macrolaimus* (Schneider, 1866) Susoy and Herrmann (2012)

*R. nacogdochensis* (Massey, 1974) Susoy and Herrmann (2012)

*R. neolongistoma* (Hussain, Tahseen, Khan and Jairajpuri, 2004) Susoy and Herrmann (2012)

*R. pellucidus* (Cobb, 1920) Susoy and Herrmann (2012)

*R. picei* (Fuchs, 1931)

*R. pini* (Fuchs, 1931)

*R. platypi* (Kanzaki, Kobayashi, Nozaki and Futai, 2006) Susoy and Herrmann (2012)

*R. rifflei* (Massey and Hinds, 1970) Susoy and Herrmann (2012)

*R. robiniae* (Harman, Winter and Harman, 2000) Susoy and Herrmann (2012)

*R. ulmi* (Goodey, 1930) Susoy and Herrmann (2012)

= *Cylindrogaster ulmi* (Goodey, 1930)

= *Rhabditolaimus schuurmansi* (Fuchs, 1933)

= *Goodeyus ulmi scolytus* (Rühm, 1956)

= *Myctolaimus ulmi* (Goodey, 1930) Sudhaus and Fürst von Lieven (2003)

*R. vitautasi* (Korenchenko, 1975) Susoy and Herrmann (2012)

*R. walkeri* (Hunt, 1980) Susoy and Herrmann (2012)

*R. zamithi* (Lordello in Andrássy, 1984) Susoy and Herrmann (2012)

#### Key to species

Tabular key to species of *Rhabditolaimus* is given in Table 4. The data were obtained from the original species descriptions and drawings. Missing values are calculated from the data in original taxonomic species descriptions and drawings, or in detailed re-descriptions.

**Table 4.** Tabular key for identification of the *Rhabditolaimus* species.

Species/characters	C1	C2	C3	C4	C5	C6	C7	C8	C9	C10	C11
*R. neolongistoma*	1	1	2	1	2	1	3	1	2	3	2
*R. platypi*	1	1	2	4	3	3	2	1	23	2	1
*R. rifflei*	1	1	3	1	2	2	1	1	3	2	3
*R. curzii*	1	1	3	1	12	3	2	2	1	2	3
*R. erectus*	1	1	3	2	2	3	1	2	1	2	2
*R. anoplophorae*	1	1	3	2	3	3	2	1	1	3	3
*R. kishtwarensis*	1	1	3	2	12	3	2	1	1	3	2
*R. pellucidus*	1	1	3	3	4	3	2	1	1	2	3
*R. macrolaimus*	1	1	3	12	1	3	2	2	1	2	3
*R. pini*	1	2	1	1	4	23	2	2	1	2	1
*R. carolinensis*	1	2	1	2	3	2	3	1	2	3	1
*R. leuckarti*	1	2	1	2	3	3	2	2	1	3	1
*R. picei*	1	2	1	2	4	3	2	1	3	3	1
*R. nacogdochensis*	1	2	1	3	3	3	1	1	3	3	1
*R. vitautasi*	1	2	2	23	3	23	2	2	1	2	2
*R. inevectus*	1	2	3	1	3	3	1	1	1	1	3
*R. zamithi*	1	3	3	2	2	2	2	1	1	1	2
*R. robiniae*	1	3	3	2	3	3	2	1	1	2	2
*R. walkeri*	1	3	3	2	3	3	2	1	1	2	3
*R. ulmi*	1	3	3	3	2	23	2	2	1	3	2
*R. goodeyi*	2	1	2	5	4	3	2	1	1	1	1
*R. dendrophilus*	2	1	3	4	4	3	3	1	1	1	2

**Notes:** Characters and species are ordered according to identification steps. Character 1. Female genital system: 1 – paired; 2 – monoprodelphic; Character 2. Male bursa: 1 – leptoderan with long spike; 2 – leptoderan with spike mucro less 10 µm; 3 – peloderan; Character 3. Ratio: stoma/corpus: 1 – 0.44 or less; 2 – 0.45 to 0.78; 3 – 0.79 or more; Character 4. Mean spicule length: 1 – less than 25 µm; 2 – 25 to 35 µm; 3 – 36 to 55 µm; 4 – 56 to 100 µm; 5 – more than 100 µm; Character 5. Female *c*′ value: 1 – more than 7; 2 – (5.1-7.0); 3 – (3.5-5.0); 4 – less than 3.5; Character 6. Male body length: 1 – less than 500 µm; 2 – 500 to 690 µm; 3 – 700 µm and longer; Character 7. Preanal male caudal papillae pairs: 1 – 2 papillae pairs; 2 – 3 papillae pairs; 3 – 4 papillae pairs; Character 8. Postanal male caudal papillae pairs: 1 – 6 papillae pairs; 2 – 7 or 8 papillae pairs; Character 9. Excretory pore position: 1 – at border isthmus and second bulb; 2 – at middle of second bulb; 3 – at pharyngeal end or posterior; Character 10. Ratio: mean corpus L/diam.: 1 – 2.5 or less; 2 – 2.6 to 3.0; 3 – more than 3.0; Character 11. Ratio: stoma/pharynx: 1 – 0.20 or less; 2 – 0.21 to 0.29; 3 – 0.3 and more.

#### Molecular characterization and phylogenetic position of the genus *Rhabditolaimus*

**Figure 10:**
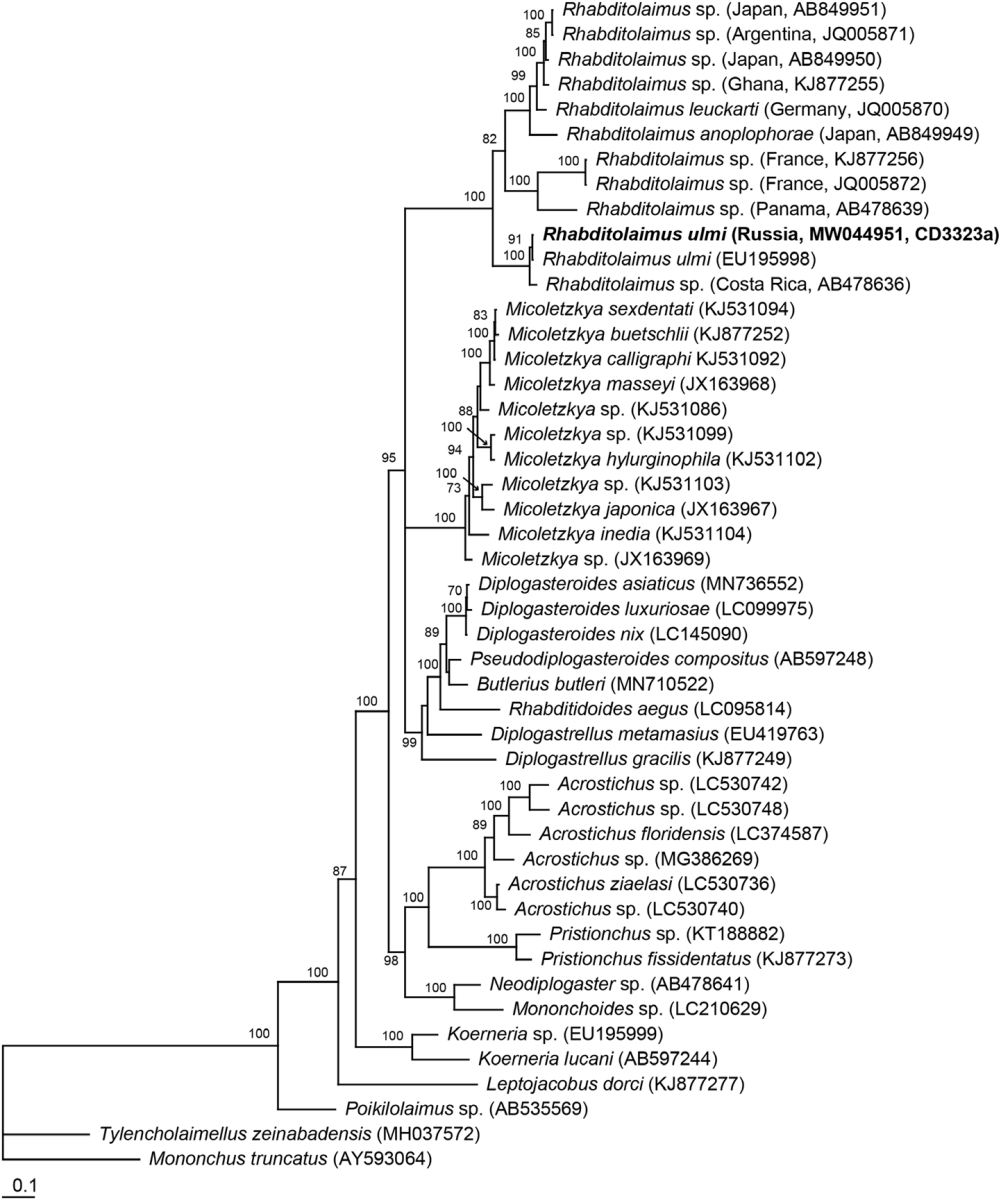
Phylogenetic relationships within *Rhabditolaimus* spp. and other related taxa as inferred from Bayesian analysis of the D2-D3 expansion segments of 28S rRNA gene sequences. Posterior probability values more than 70% are given on appropriate clades. New sequence indicated in bold.

Sequence of D2-D3 of 28S rRNA gene from *R. ulmi* was obtained in the present study. The D2-D3 of 28S rRNA gene alignment (786 bp) included 47 sequences of diplogastrids, including *R. ulmi* and two sequences of outgroup taxa. Phylogenetic analysis resulted in the Bayesian consensus tree given in Figure 10. Representatives of the genus *Rhabditolaimus* (= *Myctolaimus*) formed a highly supported clade (PP = 100%). *Rhabditolaimus ulmi* sequence from Russia (GenBank no. MW044951, CD3323a) was identical to that deposited by Kiontke et al. (2007).

## Discussion

*Rabditolaimus ulmi* is commonly found in bark of declining elms with symptoms of DED. In our seven-year survey *R. ulmi* and *B. ulmophilus* co-occurred as phoronts carried by the beetle *Scolytus multistriatus* in Northwest Russia. All developmental juvenile stages, the transmissive dauer juvenile DJ3 as well as molts between stages were studied and described in this study, except DJ2 within egg shell and DJ2-DJ3 molt. Juvenile stages of *R. ulmi* can be easily distinguished by the size, structure, and number of germinal cells of the genital primordium. Morphological differences between male and female juveniles can be diagnosed from the third-stage juvenile. According to genital primordium structure, the dauer juvenile should be considered as the third stage (DJ3). The transmissive juvenile DJ3 differs from J3 of the propogative generation by its head structure: absence of six prominent probolae, presence of hyaline head cupola instead of cuticle skeleton, cuticle stoma tube tapering into the pharyngeal capillary canal vs broad triangular canal of the stoma and pharynx in J3. The most marked feature is the small pyriform first bulb in DJ3 vs long cylindrical and well-developed corpus with a triangular lumen in J3. Additionally, the DJ3 is very slender and devoid of annulation of the surface cuticle vs broad annulated body in J3.

The shape of the first bulb (combined procorpus and metacorpus) can be used as a diagnostic character to differentiate the DJ3 and J3. The J2 inside the egg, before hatching and even in the parent female before oviposition, already has a wide cylindrical first bulb which is a characteristic feature of the propagative J2 and J3. Both J1 in an eggshell and DJ3 differ from the J2 in the compact weak first pharyngeal bulb (combined procorpus and metacorpus) vs a first bulb muscular and elongated in J2 before hatching and after hatching from an eggshell, J3 and following stages.

Researchers believe that an environmental stimulus such as exposure to the mature adult beetle in the beetle galleries or a lack of nutrition may trigger the 2nd stage juvenile to develop into a dauer (Kanzaki, 2008). It is very possible that the DJ2 within an eggshell in the female genital tube has the compact weak first pharyngeal bulb as the first bulb of J1 and DJ3. Perhaps the stimulus to shift development into the dauer stage affects the parent female, not only the juvenile itself. To confirm this hypothesis, it is will be necessary to find the J2D (predauer) of transmissive generation in the female genital tube.

The finding of a didelphic female genital system in *R. ulmi* has very important taxonomic implications in the systematics of the family Diplogastridae. Rühm (1956) in his redescription of *R. ulmi* described and illustrated monoprodelphic females. However, all females in 25 isolates we collected in St. Petersburg were didelphic. The populations we analyzed using both morphological and molecular analyses are *R. ulmi*; D2-D3 expansion segments of 28S rRNA gene sequence match a previously sequenced isolate of this species from Germany (Kiontke et al., 2007) from the same plant and insect hosts, as in the original description and in redescription (Goodey, 1930; Rühm, 1956).

The vulva position in the middle of body shown in descriptions of *R. ulmi* is typical for didelphic females and corroborates our findings; a central vulva position is typical for didelphic females as opposed to monoprodelphic females with posterior vulva. Unfortunately, the specimens used by Rühm were stained nematodes on temporary slides that cannot be examined. Type specimens of *R. ulmi* (= *Cylindrogaster ulmi* Goodey, 1930) do not exist. Later, all researchers had accepted the unpaired female genital system in *R. ulmi* as true. Here we have shown the presence of two genital branches in females of *R. ulmi*. This fact will simplify the generic systematics of the Diplogastridae, because most *Rhabditolaimus* species are didelphic with a central vulva position. Only two species of the genus: *R. goodeyi* and *R. dendrophilus* have monoprodelphic females; both species have females with the vulva posteriorly located and males with very long spicules. Probably, this feature caused by the specific position of females during copulation. Both species may be again transferred to the genus *Protocylindrocorpus* Rühm, 1959 formerly synonymized by Susoy and Herrmann (2012) with *Rhabditolaimus*. This fact may be an argument for the future study to reestablish the genus *Protocylindrocorpus*. Unfortunately, no sequencing results on both species are available in GenBank.

In our study, we propose to enumerate the male papillae first as groups and then as numbers in each group. Most of them are transversal ribs of the bursa, located anteriorly and posteriorly to the cloacal opening. Grouping of papillae according to their form and position is a perspective tool to establish homologies and thus verifies the phylogenetic analysis. Besides, the cloacal opening that used earlier as a marker of papillae positions (Kanzaki and Futai, 2004) we propose to use a phasmid position to characterize the caudal papillae pattern.

In the female genital tract, the spermatheca is not axial. It is the dorsally branching blind appendix of the uterus, indicating that the spermatheca in *Rhabditolaimus* is a section derived from the uterus and not an expanded posterior part of the oviduct (Chizhov, 1991). Sperm via a spermathecal duct is injected into the uterus; the eggshell formation and maturation of the embryo takes place also in the uterus. The uterus has no inner differentiation into sections; the preuteral gland is not discernible. However, just opposite the vagina, there is the unpaired uteral chamber. The latter is compact and bordered by two sphincters: anterior and posterior ones. This chamber is evidently used for oviposition; in many females, it contains a fully developed egg before it moves into the vagina. The uteral chamber is a special anatomical section of the genital tract not previously described in diplogastrid adult females.

Our observations reveal the combined (omnivorous) feeding of *R. ulmi*. The nematodes may feed on bacteria which they vector on their surface coat, after the bacteria multiplied in sterile PA, and the *Botryotinia fuckeliana* mycelium in PA culture. New data expand our knowledge on a range of foods of the *Rhabditolaimus* spp. (Diplogastridae), which are generally characterized only as bacterial feeders (Yeates et al., 1993).

*Rhabditolaimus ulmi* was found in bark beetle galleries; however, this species was never detected in twigs of the tree crowns injured by the maturation feeding of *S. multistraitus*. The dauers of *R. ulmi* are situated in buccal cavity of senior beetle larvae and, additionally, in the ovipositor’s cavity of beetle imago and pupae, where they adhere and remain attached also after deworming treatments, thus indicating tight relationships with the vector in comparison with the *Bursaphelenchus ulmophilus* dauers, which easily washed out from the same beetle vector after a treatment with deworming solutions.

Numerous nematode adults and juveniles were detected in excrements produced by the beetle larvae in galleries where they feed on bacteria and fungi. Evidently, that crawling nematodes fulfill the function of dispersal of the bacterial and fungal mass located in their surface coats. Further research will help to determine whether *R. ulmi* plays any role also in the pathogenesis of the Dutch elm disease, or whether its role is limited to a simple phoretic association with the beetle vectors of the causal agent of this disease. In contrast to *R. ulmi*, the co-inhabiting phoront nematode *Bursaphelenchus ulmophilus* is localized both in galleries and at the top of elm trees with symptoms of elm dieback. This indicates on its phoresy by a vector during the period when the young beetles after emergence from pupal cambers feed on young twigs to sexually mature (maturation feeding) (Santini and Faccoli, 2015). It is confirmed by the nematode dauers localization under elytra of young beetle adults (Ryss et al., 2015).

## References

[ref001] Álvarez-Ortega, S. and Peña-Santiago, R. 2016. *Aporcella charidemiensis* sp. n. (Dorylaimida: Aporcelaimidae) from the southern Iberian Peninsula, with comments on the phylogeny of the genus. Nematology 18:811–821, doi: 10.1163/15685411-00002995.

[ref002] Chizhov, V. N. 1991. “Morphology of female genital system in free-living and plant parasitic nematodes”, In Turlygina, E. S. and Chizhov, V. N. (Eds), Reproduction Biology of Plant Parasitic Nematodes Nauka Publishing, Moscow, pp. 9–57 (in Russian).

[ref003] Collins, T. J. 2007. ImageJ for microscopy. BioTechniques 43:25–30, available at: https://imagej.net. 10.2144/000112517 17936939

[ref004] Fürst von Lieven, A. and Sudhaus, W. 2000. Comparative and functional morphology of the buccal cavity of Diplogastrina (Nematoda) and a first outline of the phylogeny of this taxon. Journal of Zoological Systematics and Evolutionary Research 38:37–63, doi: 10.1046/j.1439-0469.2000.381125.x.

[ref005] Goodey, T. 1930. A new species of the nematode genus *Cylindrogaster*. Journal of Helminthology 8:89–92.

[ref006] Jones, J. T., Haegeman, A., Danchin, E. G., Gaur, H. S., Helder, J., Jones, M. G., Kikuchi, T., Manzanilla-López, R., Palomares-Rius, J. E., Wesemael, W. M. and Perry, R. N. 2013. Top 10 plant-parasitic nematodes in molecular plant pathology. Molecular Plant Pathology 14:946–961, doi: 10.1111/mpp.12057. 23809086 PMC6638764

[ref007] Kalko, G. V. 2008. Dutch elm disease in Saint Petersburg. Mikologia i Fitopatologia 6:564–571 (in Russian).

[ref008] Kalko, G. V. 2009. *Ophiostoma*-caused wilt of elms in Saint Petersburg. Zashchita i Karantin Rasteniy 3:48–49 (in Russian).

[ref009] Kanzaki and N. 2008. “Taxonomy and systematics of the nematode genus *Bursaphelenchus* (Nematoda: Parasitaphelenchinae)”, In Zhao, B. G., Futai, K., Sutherland, J. and Takeuchi, Y. (Eds), Pine Wilt Disease Springer, Tokyo, pp. 44–66.

[ref010] Kanzaki, N. and Futai, K. 2004. *Cylindrocorpus anoplophorae* n. sp. (Nematoda: Cylindrocorporidae) isolated from the white-spotted longicorn beetle, *Anoplophora malasiana* (Coleoptera: Cerambycidae). Japanese Journal of Nematology 34:11–19.

[ref011] Kanzaki, N. and Giblin-Davis, R. M. 2014. Phylogenetic status and morphological characters of *Rhabditolaimus anoplophorae* (Rhabditida: Diplogastridae). Journal of Nematology 46:44–49. 24644370 PMC3957571

[ref012] Kiontke, K., Barrière, A., Kolotuev, I., Podbilewicz, B., Sommer, R., Fitch, D. H. A. and Félix, M. -A. 2007. Trends, stasis, and drift in the evolution of nematode vulva development. Current Biology 17:1925–1937, doi: 10.1016/j.cub.2007.10.061. 18024125

[ref013] Mota, M. M., Braasch, H., Bravo, M. A., Penas, A. C., Burgermeister, W., Metge, K. and Sousa, E. 1999. First report of *Bursaphelenchus xylophilus* in Portugal and in Europe. Nematology 1:727–734, doi: 10.1163/156854199508757.

[ref014] Ronquist, F. and Huelsenbeck, J. P. 2003. MrBayes 3: Bayesian phylogenetic inference under mixed models. Bioinformatics 19:1572–1574, doi: 10.1093/bioinformatics/btg180. 12912839

[ref015] Rühm, W. 1956. Die Nematoden der Ipiden. Parasitologische Schriftenreihe 6:1–435.

[ref016] Ryss, A., Polyanina, K. S., Popovichev, B. G. and Subbotin, S. A. 2015. Description of *Bursaphelenchus* *ulmophilus* sp. n. (Nematoda: Parasitaphelenchinae) associated with Dutch elm disease of *Ulmus glabra* Huds. in the Russian North West. Nematology 17:685–703, doi: 10.1163/15685411-00002902.

[ref017] Ryss, A. Y. 2003. Express technique to prepare collection slides of nematodes. Zoosystematica Rossica 11:257–260.

[ref018] Ryss, A. Y. 2015. The most simple techniques for detection and laboratory cultivation of woody plant wilt nematodes. Izvestia Sankt-Peterburgskoj Lesotehnicheskoj Akademii 211:287–295 (in Russian).

[ref019] Ryss, A. Y. 2017a. The simplest «field» methods for extraction of nematodes from plants, wood, insects and soil, with additional description how to keep extracted nematodes alive for a long time. Parazitologiya 51:57–67. 29401577

[ref020] Ryss, A. Y. 2017b. A simple express technique to process nematodes for collection slide mounts. Journal of Nematology 49:27–32, doi: 10.21307/jofnem-22017-21043. 28512375 PMC5411252

[ref021] Ryss, A. Y., Vieira, P., Mota, M. and Kulinich, O. 2005. A synopsis of the genus *Bursaphelenchus* Fuchs, 1937 (Aphelenchida: Parasitaphelenchidae) with keys to species. Nematology 7:393–458, doi: 10.1163/156854105774355581.

[ref022] Ryss, A. Y., Parker, C., Álvarez-Ortega, S., Nadler, S. A. and Subbotin, S. A. 2021. *Bursaphelenchus juglandis* n. sp. (Nematoda: Aphelenchoididae), an associate of walnut twig beetle, *Pityophthorus juglandis*, the vector of thousand cankers disease. Nematology 23:171–, doi: 10.1163/15685411-bja10037.

[ref023] Santini, A. and Faccoli, M. 2015. Dutch elm disease and elm bark beetles: a century of association. iForest 8:126–134, doi: 10.3832/ifor1231-008.

[ref024] Susoy, V. and Herrmann, M. 2012. Validation of *Rhabditolaimus* Fuchs, 1914 (Nematoda: Diplogastridae) supported by integrative taxonomic evidence. Nematology 14:595–604, doi: 10.1163/156854111X617419.

[ref025] Susoy, V., Ragsdale, R. J., Kanzaki, N. and Sommer, R. J. 2015. Rapid diversification associated with a macroevolutionary pulse of developmental plasticity. eLife 2015:e05463, doi: 10.7554/eLife.05463. PMC4357287 25650739

[ref026] Tanha Maafi, Z., Subbotin, S. A. and Moens, M. 2003. Molecular identification of cyst-forming nematodes (Heteroderidae) from Iran and a phylogeny based on the ITS sequences of rDNA. Nematology 5:99–111, doi: 10.1163/156854102765216731.

[ref027] Yeates, G. W., Bongers, T., De Goede, R. G. M., Freckman, D. W. and Georgieva, S. S. 1993. Feeding habits in soil nematode families and genera--an outline for soil ecologists. Journal of Nematology 25:315–331, PMCID: PMC2619405. 19279775 PMC2619405

